# Additions to *Rhytidhysteron* (*Hysteriales*, *Dothideomycetes*) in China

**DOI:** 10.3390/jof9020148

**Published:** 2023-01-22

**Authors:** Tian-Ye Du, Dong-Qin Dai, Ausana Mapook, Li Lu, Steven L. Stephenson, Nakarin Suwannarach, Abdallah M. Elgorban, Salim Al-Rejaie, Samantha C. Karunarathna, Saowaluck Tibpromma

**Affiliations:** 1 Center for Yunnan Plateau Biological Resources Protection and Utilization, College of Biological Resource and Food Engineering, Qujing Normal University, Qujing 655011, China; 2 Center of Excellence in Fungal Research, Mae Fah Luang University, Chiang Rai 57100, Thailand; 3 School of Science, Mae Fah Luang University, Chiang Rai 57100, Thailand; 4 Department of Biological Sciences, University of Arkansas, Fayetteville, AR 72701, USA; 5 Research Center of Microbial Diversity and Sustainable Utilization, Chiang Mai University, Chiang Mai 50200, Thailand; 6 Department of Botany and Microbiology, College of Science, King Saud University, Riyadh 11451, Saudi Arabia; 7 Department of Pharmacology & Toxicology, College of Pharmacy, King Saud University, Riyadh 11451, Saudi Arabia

**Keywords:** *Ascomycota*, four new species, *Hysteriaceae*, hysteriaceous, saprobes, seven new records

## Abstract

In this study, twelve terrestrial hysteriaceous saprobic fungi growing on different pieces of dead wood were collected from Yunnan Province, China. All hysteriaceous strains isolated in this study tallied with the general characteristics associated with *Rhytidhysteron*. Detailed morphological characteristics and combined multigene phylogeny of LSU, ITS, SSU, and *TEF* showed that the twelve hysteriaceous fungi strains represent four distinct new species, and seven new host or geographical records of *Rhytidhysteron*. Based on morphological and phylogenetic evidence, the four new species (*Rhytidhysteron bannaense* sp. nov., *R. coffeae* sp. nov., *R. mengziense* sp. nov., and *R. yunnanense* sp. nov.) expand the number of species of *Rhytidhysteron* from thirty-three to thirty-seven, while seven new geographical records expand the records of *Rhytidhysteron* in China from six to thirteen. In addition, 10 new *Rhytidhysteron* host records are reported for the first time, thus expanding the known hosts for *Rhytidhysteron* from 52 to 62. Full descriptions, images of the morphology, and phylogenetic analyses to show the position of the *Rhytidhysteron* taxa are provided. In addition, the present study summarizes the main morphological characteristics, host associations, and locations of this genus.

## 1. Introduction

The *Dothideomycetes* O.E. Erikss. & Winka is the largest class in the *Ascomycota* Caval.-Sm [1,2,3]. Currently, it is made up of the *Dothideomycetidae* P.M. Kirk, P.F. Cannon, J.C. David & Stalpers (three orders with 25 families), and *Pleosporomycetidae* C.L. Schoch, Spatafora, Crous & Shoemaker (four orders with 94 families) [2,4,5]. This highly diverse class is mainly characterized by bitunicate asci (asci with two-wall layers), and often with fissitunicate dehiscence [2,6].

The *Hysteriales* Lindau belong to the subclass *Pleosporomycetidae* [4,5]. This monotypic order is characterized by its thick-walled, navicular ascomata which dehisce by an invaginated slit or sulcus [4,7].

The *Hysteriaceae* Chevall., the only family in *Hysteriales*, has been classified under the *Pseudosphaeriales* E. Müll. & Arx, *Dothiorales* Luttrell, *Dothideales* Lindau, and the *Pleosporales* Luttr. ex M.E. Barr, previously [4,8,9,10,11,12]. The *Hysteriaceae* include the hysteriaceous fungi, which currently contain 13 genera [5]. Hysteriaceous fungi are characterized by hysterithecioid or apothecioid ascomata, semi-immersed to superficial, carbonaceous, thick-walled, and distinctly navicular with a pronounced, longitudinal slit, ascospores that are hyaline to pigmented, muriform, and one to multi-septate in bitunicate asci [2,12,13,14,15,16,17,18] 

The genus *Rhytidhysteron* Speg. was introduced by Spegazzini [19] to accommodate two species (*R*. *brasiliense* Speg. and *R. viride* Speg.), but no type species was designated. Subsequently, Clements and Shear [20] designated *R. brasiliense* as the type species [12,16,21,22]. The genus was transferred by Boehm et al. [13] from the *Patellariaceae* Corda to *Hysteriaceae,* based on molecular data. Currently, 33 records of *Rhytidhysteron* are listed in the Index Fungorum [23]. The sexual morph is described as having large ascomata, conspicuous, navicular, usually with a perpendicular striae margin, and pigmented, septate and muriform to sub-muriform ascospores [16,18]. Currently, only four species (*R. hysterinum* [Dufour] Samuels & E. Müll., *R. rufulum* [Spreng] Speg., *R. thailandicum* Thambug. & K.D. Hyde, and *R. xiaokongense* G.C. Ren & K.D. Hyde) have been described with an asexual morph, and conidia are classified into two types—“*Aposphaeria*-like” and “*Diplodia*-like” [16,18,21]. The main characteristics used to distinguish some species in this genus are the shape and border of the hysterothecium, the type of the exciple, the color and reaction of the epithecium, and the size of the ascospores [24]. Species of *Rhytidhysteron* are widely distributed in 33 countries and on 52 hosts [6,18,25,26]. Members of *Rhytidhysteron* play an indispensable role as saprobes, endophytes, and weak pathogens on woody plants in both terrestrial and marine habitats, and some are rarely found as human pathogens [6,16,18,25,27]. 

In this study, we collected 12 strains of hysteriaceous fungi from Yunnan Province, China, and based on molecular phylogenetic analyses (LSU, ITS, SSU, and *TEF*) and morphological characteristic comparisons, they were identified as four distinct new species (*Rhytidhysteron bannaense* sp. nov., *R. coffeae* sp. nov., *R. mengziense* sp. nov., and *R. yunnanense* sp. nov.) and seven new host records (*R. bruguierae* Dayar., *R. camporesii* Ekanayaka & K.D. Hyde, *R. hongheense* Wanas., *R. magnoliae* N.I. de Silva & K.D. Hyde, *R. neorufulum* Thambug. & K.D. Hyde, *R. tectonae* Doilom & K.D. Hyde, and *R. thailandicum*).

## 2. Materials and Methods

### 2.1. Sample Collection, Morphological Identification, and Single Spore Isolation

Dead plant specimens with fungal fruiting bodies were collected from Yunnan Province, China, between 2020 and 2021. Specimens were placed in plastic bags, important information such as collection date, location, and host name was recorded, and then the specimens were brought them back to the lab for isolation and morphological observation. 

Senanayake et al. [28] was followed for the morphological study and single spore isolation. Morphological structures were examined under an OPTEC SZ650 dissecting stereomicroscope. An OLYMPUS optical microscope (Tokyo, Japan) was used to observe microscopic fungal structures and an OLYMPUS DP74 (Tokyo, Japan) digital camera fitted to the microscope was used to take photographs. All micro-morphological structures were measured with the Tarosoft ^®^ Image Framework program and photo plates were made using Adobe Photoshop CS3 Extended version 10.0 software (Adobe Systems, San Jose, CA, USA).

Single spore isolation was carried out for all the specimens and the pure cultures were grown on potato dextrose agar (PDA). Germinated spores were transferred to new PDA plates under sterile conditions and incubated at 28 °C. Culture characteristics (mycelia color, shape, and edge feature) were observed after one week. 

The specimens were deposited in the Kunming Institute of Botany, Academia Sinica (HKAS), Kunming, China. Living cultures were deposited in the Kunming Institute of Botany Culture Collection (KUMCC), China. Facesoffungi (FoF) numbers were registered as described in Jayasiri et al. [29], and MycoBank number (MB) was registered as outlined in MycoBank (http://www.MycoBank.org, accessed on 24 October 2022).

### 2.2. DNA Extraction, PCR Amplification, and Sequencing

Dissanayake et al. [30] was followed for molecular studies. Fresh mycelia which grew on PDA plates for two weeks were scraped off the plates, and then DNA was extracted using DNA Extraction Kit-BSC14S1 (BioFlux, Hangzhou, P.R. China), following the manufacturer’s protocol. Polymerase chain reaction (PCR) was used to amplify four gene regions and the primers and protocols were used for the amplification following Wanasinghe et al. [25]. The LSU gene was amplified by using the primers LR0R and LR5 [31], the ITS gene was amplified by using the primers ITS5 and ITS4 [32], the SSU gene was amplified using the primers NS1 and NS4 [32], and the *TEF* gene was amplified using the primers EF1-983F and EF1-2218R [33]. The total volume of the PCR mixture for amplifications was 25 μL, which consisted of 12.5 μL 2xMaster Mix (mixture of Easy Taq TM DNA Polymerase, dNTPs, and optimized buffer (Beijing Trans Gen Biotech Co., Chaoyang District, Beijing, China)), 8.5 μL ddH_2_O, 2 μL of DNA template, and 1 μL of each forward and reverse primer (10 pM) [34]. Purification and sequencing of PCR products were carried out by Qinke Biotech Co., Kunming, China. 

### 2.3. Phylogenetic Analyses

The sequences of all strains obtained in this study were checked in BioEdit v.7.2.6.1 [35] for quality. Geneious 9.1.8 was used to splice forward and reverse sequences. The combined sequences were searched for similar taxa via Blast in NCBI (http://blast.ncbi.nlm.nih.gov/ (accessed on 10 November 2022)), and the most closely related taxa were put together for the phylogenetic analyses. Phylogenetic analyses were carried out with 65 sequences (Table 1) and two outgroup taxa—*Gloniopsis calami* S. Konta & K.D. Hyde (MFLUCC 15-0739) and *G. praelonga* (Schwein.) Underw. & Earle (CBS 112415). All four gene sequences were downloaded from NCBI (http://www.ncbi.nlm.nih.gov/ (accessed on 10 November 2022)) and aligned by MAFFT v.7 (http://mafft.cbrc.jp/alignment/server/ (accessed on 10 November 2022)) [36]. TrimAl.v1.2rev59 was used to optimize the alignment of sequences [37], sequences were combined in BioEdit v.7.2.6.1. FASTA alignment formats were converted to PHYLIP and NEXUS formats in ALTER (http://www.sing-group.org/ALTER/ (accessed on 10 November 2022)) [38]. 

In phylogenetic analyses, Randomized Accelerated Maximum Likelihood (RAxML) and Bayesian inference analyses (BI) were carried out in the CIPRES Science Gateway (https://www.phylo.org/portal2/login!input.action (accessed on 10 November 2022)) [39]. The RAxML trees are performed using RAxML-HPC2 on XSEDE (8.2.12) [40,41] with the GTR + I + G model of evolution. Additionally, Bayesian analyses were conducted using the Markov Chain Monte Carlo (MCMC) method in MrBayes on XSEDE (3.2.7a) [42] to evaluate posterior probabilities [43,44]: the best model of LSU and ITS is GTR + I+G, the best model of SSU is HKY + I, and the best model of *TEF* is GTR + G. Six simultaneous Markov chains were run for 1,000,000 generations, and trees were sampled at every 100th generation. Max-trees was set to 5000 and clade robustness was assessed using a bootstrap (BT) analysis of 1000 replicates. Phylogenetic trees were visualized with FigTree v.1.4.2 [45], bootstrap values showing at the nodes, and edited by Microsoft Office PowerPoint 2010. The newly obtained alignments and phylogenetic trees were deposited in TreeBASE, submission ID: 30049 (https://treebase.org/treebase-web/user/submissionList.html, accessed on 28 November 2022).

## 3. Results

### 3.1. Phylogenetic Analyses

**Figure 1:**
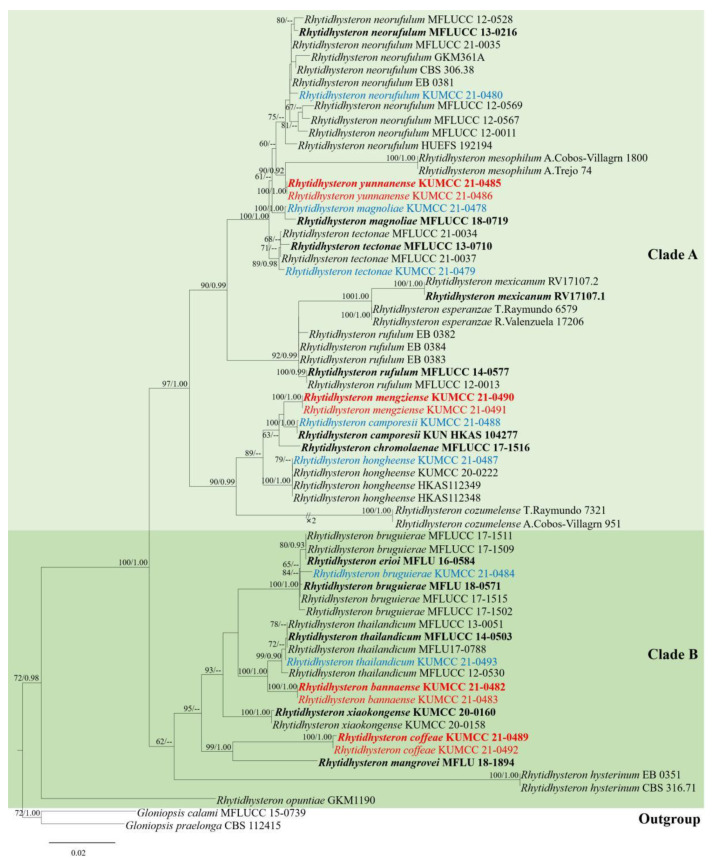
Phylogenetic tree generated from RAxML analyses based on combined LSU, ITS, SSU, and *TEF* sequence data for *Rhytidhysteron*. The tree was rooted with *Gloniopsis calami* (MFLUCC 15-0739) and *G. praelonga* (CBS 112415). Bootstrap support values equal to or higher than 60% ML and posterior probability values equal to or higher than 0.90 Bayesian PP are indicated on the nodes. New species are in red, new records are in blue, and ex-type strains are in bold.

The phylogenetic trees obtained from RAxML and BI analyses provided essentially similar topologies. The RAxML analyses of the combined dataset yielded the best scoring tree (Figure 1), with a final ML optimization likelihood value of −12081.382479. The matrix had 761 distinct alignment patterns, with 23.87% being undetermined characters or gaps. Parameters for the GTR + I + G model of the combined LSU, ITS, SSU, and *TEF* were as follows: estimated base frequencies A = 0.239683, C = 0.247793, G = 0.276186, T = 0.236338; substitution rates AC = 1.225201, AG = 2.493063, AT = 1.186723, CG = 0.732384, CT = 4.977839, GT = 1.0; proportion of invariable sites I = 0.675821; and gamma distribution shape parameter α = 0.554013. The final RAxML tree is shown in Figure 1.

The final RAxML tree was divided into two clades of *Rhytidhysteron* and the results are similar to those reported by Wanasinghe et al. [25]. In this study, two new species *R. mengziense* (KUMCC 21-0490, KUMCC 21-0491) and *R. yunnanense* (KUMCC 21-0485, KUMCC 21-0486), and five new records for *R. camporesii* (KUMCC 21-0488), *R. hongheense* (KUMCC 21-0487), *R. magnoliae* (KUMCC 21-0478), *R. neorufulum* (KUMCC 21-0480), and *R. tectonae* (KUMCC 21-0479) were clustered within clade A. Two new species *R. bannaense* (KUMCC 21-0482, KUMCC 21-0483) and *R. coffeae* (KUMCC 21-0489, KUMCC 21-0492), along with two new records, *R. bruguierae* (KUMCC 21-0484) and *R. thailandicum* (KUMCC 21-0493), were included in clade B. 

The four new species formed separate branches in the phylogenetic tree. *Rhytidhysteron bannaense* was well separated from *R. thailandicum* in an independent lineage with relatively good statistical support (100% ML/1.00 PP). *Rhytidhysteron coffeae* was separated from *R. mangrovei* Vinit & K.D. Hyde with good statistical support (99% ML/1.00 PP). *Rhytidhysteron mengziense* was well separated from *R. camporesii* with low statistical support. *Rhytidhysteron yunnanense* was separated from *R. mesophilum* Cobos-Villagrán, R. Valenz, Hern.-Rodr., Calvillo-Medina & Raymundo, with good statistical support (90% ML/0.92 PP). 

The seven newly recorded strains and the known species in *Rhytidhysteron* clustered together in the phylogenetic tree with significant statistical support. *Rhytidhysteron bruguierae* (KUMCC 21-0484) grouped within five strains of *R. bruguierae*, with moderate statistical support (65% ML). *Rhytidhysteron camporesii* (KUMCC 21-0488) grouped with *R. camporesii* (KUN-HKAS 104277) with good statistical support (100% ML/1.00 PP). *Rhytidhysteron hongheense* (KUMCC 21-0487) grouped with *R. hongheense* (KUMCC 20-0222, H KAS112348, HKAS112349) with moderate statistical support (79% ML). *Rhytidhysteron magnoliae* (KUMCC 21-0478) grouped with *R. magnoliae* (MFLUCC 18-0719) with good statistical support (100% ML/1.00 PP). *Rhytidhysteron neorufulum* (KUMCC 21-0480) grouped with ten strains of *R. neorufulum* with low statistical support. *Rhytidhysteron tectonae* (KUMCC 21-0479) grouped with *R. tectonae* (MFLUCC 13-0710, MFLUCC 21-0037, MFLUCC 21-0034) with good statistical support (89% ML/0.98 PP). *Rhytidhysteron thailandicum* (KUMCC 21-0493) grouped within four strains of *R. thailandicum* with moderate statistical support (72% ML). 

### 3.2. Taxonomy

**Figure 2:**
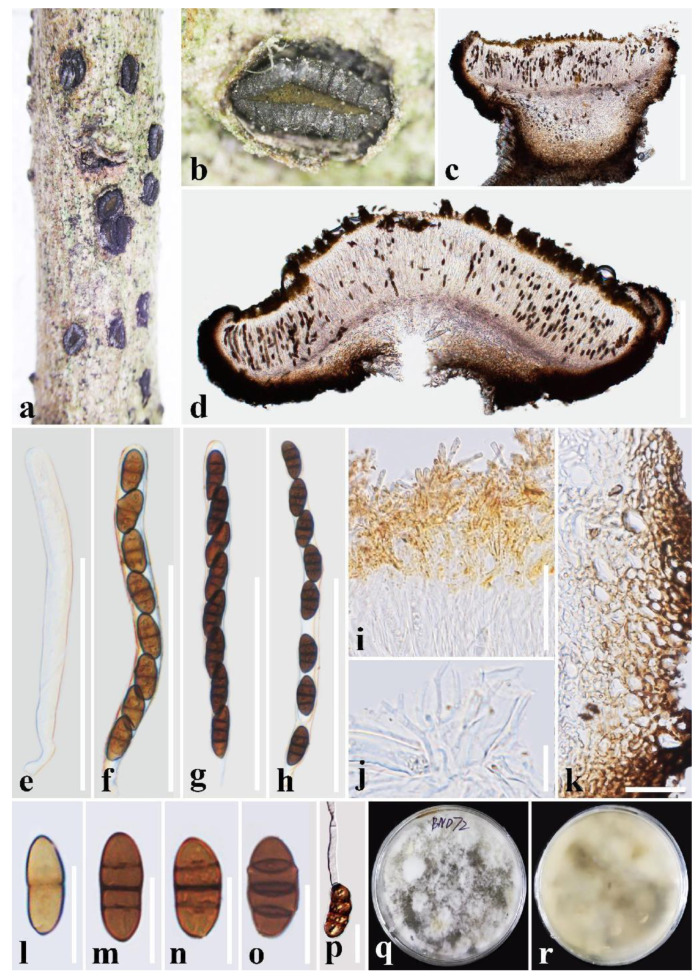
***Rhytidhysteron bannaense*** (HKAS 122695, **holotype**). (**a**,**b**) Appearance of hysterothecia on the host; (**c**,**d**) Vertical section through hysterothecium; (**e**–**h**) Asci; (**i**) Epithecium mounted in water; (**j**) Pseudoparaphyses; (**k**) Exciple; (**l**–**o**) Ascospores; (**p**) A germinating ascospore; (**q**,**r**) Colony on PDA medium (after four weeks). Scale bars: (**c**,**d**) = 500 μm; (**e**–**h**) = 100 μm; (**i**,**j**,**l**–**p**) = 20 μm; (**k**) = 50 μm.

***Rhytidhysteron bannaense*** T.Y. Du and Tibpromma sp. nov. (Figure 2)

MycoBank number: MB 845999. Facesoffungi number: FoF 12957

Etymology: Named after the region Xishuangbanna where the type specimen of the new species was collected.

Holotype: HKAS 122695

*Saprobic* on decaying wood of *Buddleja officinalis* (Loganiaceae). Sexual morph: *Ascomata*. 1000–1500 μm long × 550–1100 μm wide × 350–850 μm high (x = 1350 × 750 × 670 μm, n = 5), hysterothecial, solitary to aggregated, semi-immersed to superficial, black, apothecioid, navicular, rough, perpendicular striae, elongate and depressed, compressed at apex, opening through a longitudinal slit, green at the center. *Exciple*: 40–150 μm wide, composed of dark brown, thick-walled cells of *textura angularis*, outer layer brown to dark brown, inner layer pale brown to hyaline. *Hamathecium*: 1–2 μm wide, dense, hyaline, septate, branched, cellular pseudoparaphyses, forming an orange epithecium above asci when mounted in water, becoming a purple epithecium above the asci when mounted in 10% KOH and turns hyaline after 5 s. *Asci*: 140–189(–199) × 12–15(–16) μm (x = 166 × 14 μm, n = 20), 8-spored, bitunicate, cylindrical, with short pedicel, rounded at the apex, with an ocular chamber, J- apical ring. *Ascospores*: 22–27 × 10–12.8 μm (x = 25 × 11.5 μm, n = 30), uni-seriate, slightly overlapping, hyaline, 1-septate when immature, becoming brown to dark brown, 3-septate when mature, ellipsoidal, rounded to slightly pointed at both ends, smooth-walled, without guttules or mucilaginous sheath. Asexual morph: Undetermined.

Culture characteristics: Ascospores germinated on PDA within 24 h and germ tubes produced from one or both ends. Colonies on PDA reached a 6 cm diameter after two weeks at 28 °C. The colony is soft, circular, irregularly raised, with an undulated edge, white to gray on the forward, and grayish yellow in reverse. 

Material examined: China, Yunnan Province, Xishuangbanna Prefecture, Jinghong City, Manlie Village, 101°0′1″ E, 21°55′15″ N, on a decaying wood piece of *Buddleja officinalis* Maxim. (Loganiaceae), 12 September 2021, T.Y. Du, BND72 (holotype, HKAS 122695, ex-type living culture, KUMCC 21-0482 = KUMCC 21-0483).

Notes: In phylogenetic analyses, *Rhytidhysteron bannaense* was well separated from *R. thailandicum* with relatively good statistical support (100% ML/1.00 PP). In morphology, *R. bannaense* is distinct from *R. thailandicum*, having 8-spored asci, and ascospores 3-septate when mature, without guttules, while *R. thailandicum* has (3–)6–8-spored asci, (1–)3-septate ascospores, and guttulate [6,16,54]. In addition, the asci and ascospore size in *R. bannaense* are larger than those in *R. thailandicum* (asci: 166 × 14 μm vs. 145 × 12.8 μm; ascospores: 25 × 11.5 μm vs. 24.5 × 9.5 µm) [16]. Moreover, according to the comparison results of different gene fragments (Table 2), *R. bannaense* is different from *R*. *thailandicum* in ITS and *TEF* genes (≥1.5%, without gaps). Therefore, in this study, *R. bannaense* is introduced as a new species. 

***Rhytidhysteron bruguierae*** Dayarathne, Mycosphere 11(1): 20 (2020) (Figure S1)

MycoBank number: MB 556574. Facesoffungi number: FoF 06154

Description: See Dayarathne et al. [47], Supplementary Notes 1

Distribution: China (this study), Thailand [47,48]. 

Host: *Alnus nepalensis* (this study), *Bruguiera* sp. [47], *Chromolaena odorata* [48].

Material examined: China, Yunnan Province, Xishuangbanna Prefecture, Jinghong City, Manlie Village, 101°1′1″ E, 21°54′0″ N, on decaying wood of *Alnus nepalensis* D. Don (Betulaceae), 12 September 2021, T.Y. Du, BND77 (HKAS 122690, living culture, KUMCC 21-0484).

Notes: *Rhytidhysteron bruguierae* was introduced by Dayarathne et al. (2020) based on both morphology and phylogenetic analyses. According to phylogenetic analyses based on combined multi-gene (LSU, ITS, SSU, and *TEF*), our collection grouped together with *R. bruguierae* (MFLUCC 18-0398, MFLUCC 17-1515, MFLUCC 17 1511, MFLUCC 17-1502, MFLUCC 17-1509). The morphological characteristics of our collection resemble *R. bruguierae* in having superficial, perpendicular striae, orange at the center hysterothecia, hamathecium comprising septate, branched pseudoparaphyses, forming a red epithecium above the asci, cylindrical, short pedicellate asci, and ellipsoidal to fusiform, brown ascospores [47,48]. Our collection and *R. bruguierae* are extremely similar in molecular data analyses and morphological characteristics. Previously, this species was only recorded in Thailand from *Bruguiera* sp. and *Chromolaena odorata* [47,48]. Therefore, our collection was introduced as a new geographical and host record of *R. bruguierae* from the decaying wood of *Alnus nepalensis* (Betulaceae) in the Yunnan Province of China. This is the first record of *R. bruguierae* on *Alnus nepalensis*.

***Rhytidhysteron camporesii*** Ekanayaka & K.D. Hyde, Fungal Diversity 100: 5–277 (2020) (Figure S2)

MycoBank number: MB 556783. Facesoffungi number: FoF 06459

Description: See Hyde et al. [49], Supplementary Notes 2

Distribution: China [49] (this study).

Host: *Cotoneaster franchetii* (this study), unidentified wood [49].

Material examined: China, Yunnan Province, Kunming City, Panlong District, Changchong Mountain, on decaying wood of *Cotoneaster franchetii* Bois (Rosaceae), 5 September 2021, T.Y. Du, KMD93 (HKAS 122698, living culture, KUMCC 21-0488).

Notes: *Rhytidhysteron camporesii* was introduced by Hyde et al. [49] based on both morphology and phylogenetic analyses. According to phylogenetic analyses based on combined multi-gene (LSU, ITS, SSU, and *TEF*), our collection grouped together with *R. camporesii* (KUN-HKAS 104277). In addition, our collection shows similar morphological characteristics with *R. camporesii*, having black, striated hysterothecia, branched pseudoparaphyses, 8-spored, cylindrical, short pedicellate asci, and 3-septate, uni-seriate, ellipsoidal to fusiform, brown ascospores [49]. Therefore, we report our collection as a new host record of *R. camporesii* from decaying wood of *Cotoneaster franchetii* (Rosaceae) in the Yunnan Province of China. This is the first record of *R. camporesii* on *Cotoneaster franchetii*. 

**Figure 3:**
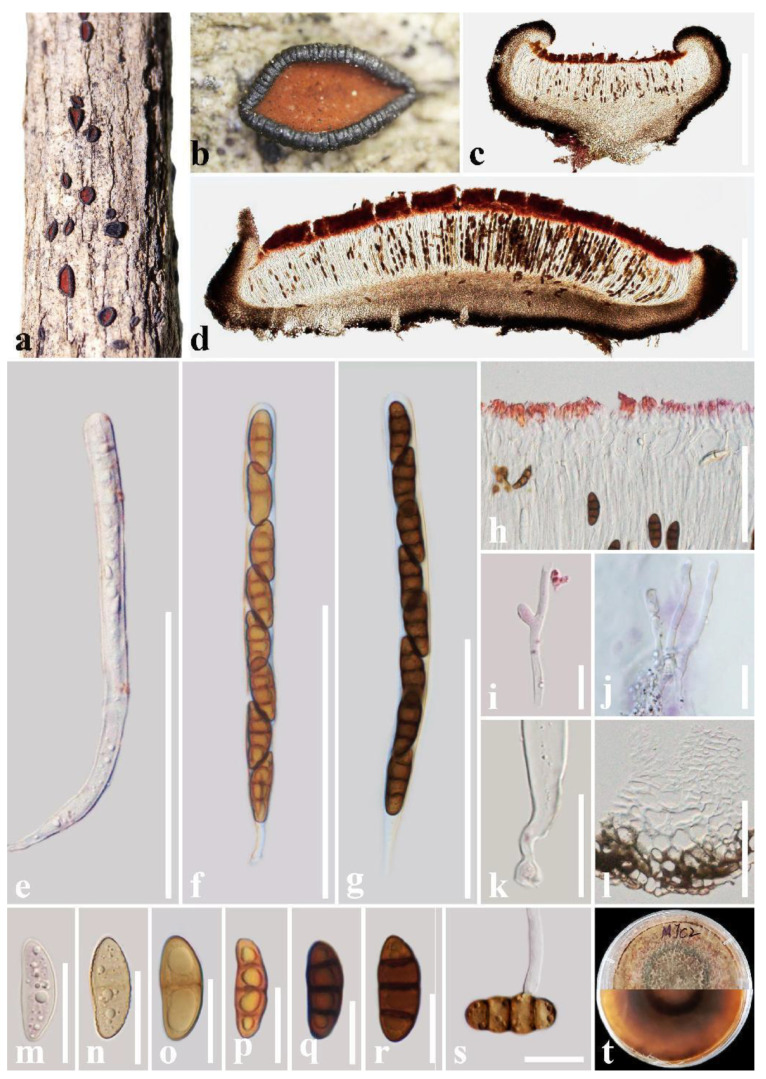
***Rhytidhysteron coffeae*** (HKAS 122701, **holotype**). (**a**,**b**) Appearance of hysterothecia on the host; (**c**,**d**) Vertical section through hysterothecium; (**e**–**g**) Asci; (**h**) Epithecium mounted in water; (**i**,**j**) Pseudoparaphyses; (**k**) Pedicel of asci; (**l**) Exciple; (**m**–**r**) Ascospores; (**s**) A germinating ascospore; (**t**) Colony on PDA medium (after four weeks). Scale bars: (**c**,**d**) = 500 μm; (**e**–**g**) = 100 μm; (**h**,**l**) = 50 μm; (**i**–**k**,**m**–**s**) = 20 μm.

***Rhytidhysteron coffeae*** T.Y. Du and Tibpromma sp. nov. (Figure 3)

MycoBank number: MB 846000. Facesoffungi number: FoF 12958

Etymology: Named after the host name, *Coffea* sp.

Holotype: HKAS 122700

*Saprobic* on decaying wood of *Coffea* sp. (Rubiaceae). Sexual morph: *Ascomata* 1000–1700 μm long × 1000–1200 μm wide × 300–600 μm high (x = 1520 × 1120 × 450 μm, n = 5), hysterothecial, solitary to aggregated, mostly solitary, superficial, base is embedded in the plant tissue, navicular, black, apothecioid, rough, perpendicular striae, elongate and depressed, compressed at apex, and opening through a nearly circular longitudinal slit, reddish brown at the center. *Exciple*: 70–160 μm wide (x = 95 μm, n = 10), composed of dark brown, thick-walled cells of *textura angularis*, outer layer brown to dark brown, inner layer pale brown to hyaline. *Hamathecium*: 2–3 μm wide, dense, hyaline, septate, branched, cellular pseudoparaphyses, forming a red to purple epithecium above asci when mounted in water, becoming dark purple epithecium above the asci when mounted in 10% KOH, and turns hyaline after 5 s. *Asci*: (162–)170–197 μm × (9–)10–14(–16) μm (x = 179.5 × 13 μm, n = 20), 8-spored, bitunicate, cylindrical, with short pedicel, rounded at the apex, with an ocular chamber, and J- apical ring. *Ascospores*: 23–28.5 μm × 8.5–11.5 μm (x = 26 × 10 μm, n = 30), uni-seriate, slightly overlapping, hyaline, 1-septate when immature, becoming reddish brown to brown, 3-septate when mature, ellipsoidal to fusoid, straight or curved, rounded to slightly pointed at both ends, guttulate, smooth-walled, without a mucilaginous sheath. Asexual morph: Undetermined.

Culture characteristics: Ascospores germinated on PDA within 24 h and germ tubes produced from one or both ends. Colonies on PDA reached a 6 cm diameter after two weeks at 28 °C. The colony is flossy, velvety, circular, slightly raised, with an entire edge, reddish brown on the forward and in reverse, with a green circle in the middle. 

Material examined: China, Yunnan Province, Pu’er City, Mojiang County, Jinggong coffee plantation, 101°44′20″ E, 23°15′15″ N, decaying wood of *Coffea* sp. L. (Rubiaceae), 23 December 2020, L. Lu, MJC2 (holotype, HKAS 122700, ex-type living culture, KUMCC 21-0489); Yunnan Province, Pu’er City, Qixiang coffee plantation, 101°20′47″ E, 22°42′15″ N, on decaying wood of *Coffea* sp. (Rubiaceae), 25 December 2020, L. Lu, QXC8 (HKAS 122701 paratype, ex-paratype culture, KUMCC 21-0492).

Notes: In the phylogenetic analyses, *Rhytidhysteron coffeae* clearly separated from *R. mangrovei* with good statistical support (99% ML/1.00 PP). With respect to morphology, *R. coffeae* is distinct from *R. mangrovei* in having branched pseudoparaphyses, 8-spored asci, and 3-septate ascospores when mature, while *R. mangrovei* has unbranched pseudoparaphyses, (2–6–)8-spored asci, and (1–)3-septate ascospores [12]. In addition, the ascomata, asci, and ascospore size of *R. coffeae* are larger than those of *R. mangrovei* (ascomata:1520 × 1120 × 450 μm vs. 940 × 800 × 500 μm, asci: 179.5 × 13 μm vs. 146 × 9.5 μm, ascospores: 26 × 10 μm vs. 23 × 8.3 μm) [12]. Moreover, according to the comparison results of different gene fragments, *R. coffeae* is different from *R. mangrovei* in ITS (77/651 bp, 11.83%, without gaps) and *TEF* (21/960 bp, 2.19%, without gaps) genes (≥1.5%). Therefore, in this study, *R. coffeae* is introduced as a new species. 

***Rhytidhysteron hongheense*** Wanas. J. Fungi 7, 180 (2021) (Figure S3)

MycoBank number: MB 837992

Description: See Wanasinghe et al. [25], Supplementary Notes 3

Distribution: China [25] (this study).

Host: *Dodonaea* sp. [25], *Phyllanthus emblica* (this study).

Material examined: China, Yunnan Province, Kunming City, Panlong District, Kunming Institute of Botany, 102°45′5″ E, 25°8′30″ N, on decaying wood of *Phyllanthus emblica* (Euphorbiaceae), 1 February 2021, T.Y. Du, KMD24 (HKAS 122697, living culture, KUMCC 21-0487).

Notes: *Rhytidhysteron hongheense* was introduced by Wanasinghe et al. [25] based on both morphology and phylogenetic analyses. According to phylogenetic analyses based on combined multi-gene (LSU, ITS, SSU, and *TEF*), our collection grouped together with *R. hongheense* (KUMCC 20-0222, HKAS112348, and HKAS112349). In addition, our collection shows similar morphological characteristics with *R. hongheense* in having solitary to aggregated, slightly striated hysterothecia, branched pseudoparaphyses forming a red epithecium above asci, 8-spored, cylindrical, short pedicellate asci, and uni-seriate ascospores, brown when mature [25]. Therefore, we report our collection as a new host record of *R. hongheense* from decaying wood of *Phyllanthus emblica* (Euphorbiaceae) in the Yunnan Province of China. This is the first record of *R. hongheense* on *Phyllanthus emblica*. 

***Rhytidhysteron magnoliae*** N.I. de Silva, Lumyong S & K.D. Hyde, Asian Journal of Mycology 3(1): 295–306 (2019) (Figure S4)

MycoBank number: MB 557220. Facesoffungi number: FoF 07369

Description: See de Silva et al. [6], Supplementary Notes 4

Distribution: China [6] (this study).

Host: *Hevea brasiliensis* (this study), *Magnolia grandiflora* [6].

Material examined: China, Yunnan Province, Xishuangbanna Prefecture, Mengla County, Xishuangbanna Tropical Botanical Garden, 101°15′40″ E, 21°55′57″ N, on decaying wood of *Hevea brasiliensis* (Willd. ex A. Juss.) Muell. Arg. (Euphorbiaceae), 24 November 2020, T.Y. Du, BND10 (HKAS 122693, living culture, KUMCC 21-0478).

Notes: *Rhytidhysteron magnoliae* was introduced by de Silva et al. [6] based on both morphology and phylogenetic analyses. According to phylogenetic analyses based on combined multi-gene (LSU, ITS, SSU, and *TEF*), our collection grouped together with *R. magnoliae* (MFLUCC 18-0719). In addition, our collection shows similar morphological characteristics to *R. magnoliae* in having solitary to aggregated, semi-immersed to superficial, coriaceous, striated hysterothecia, septate pseudoparaphyses slightly swollen at the apex and enclosed in a gelatinous matrix, 8-spored, cylindrical, short pedicellate asci, and ellipsoidal to fusoid, 1–3-septate, guttulate, brown to dark brown ascospores [6]. There is a small difference between our collection and *R. magnoliae* in that the perpendicular striae of ascomata in our collection is not as obvious as that in *R. magnoliae*. However, in the phylogenetic tree, our collection (KUMCC 21-0478) grouped with *R. magnoliae* (MFLUCC 18-0719) (100% ML/1.00 PP). Therefore, we report our collection as a new host record of *R. magnoliae* from decaying wood of *Hevea brasiliensis* (Euphorbiaceae) in the Yunnan Province of China. This is the first record of *R. magnoliae* on *Hevea brasiliensis*. 

**Figure 4:**
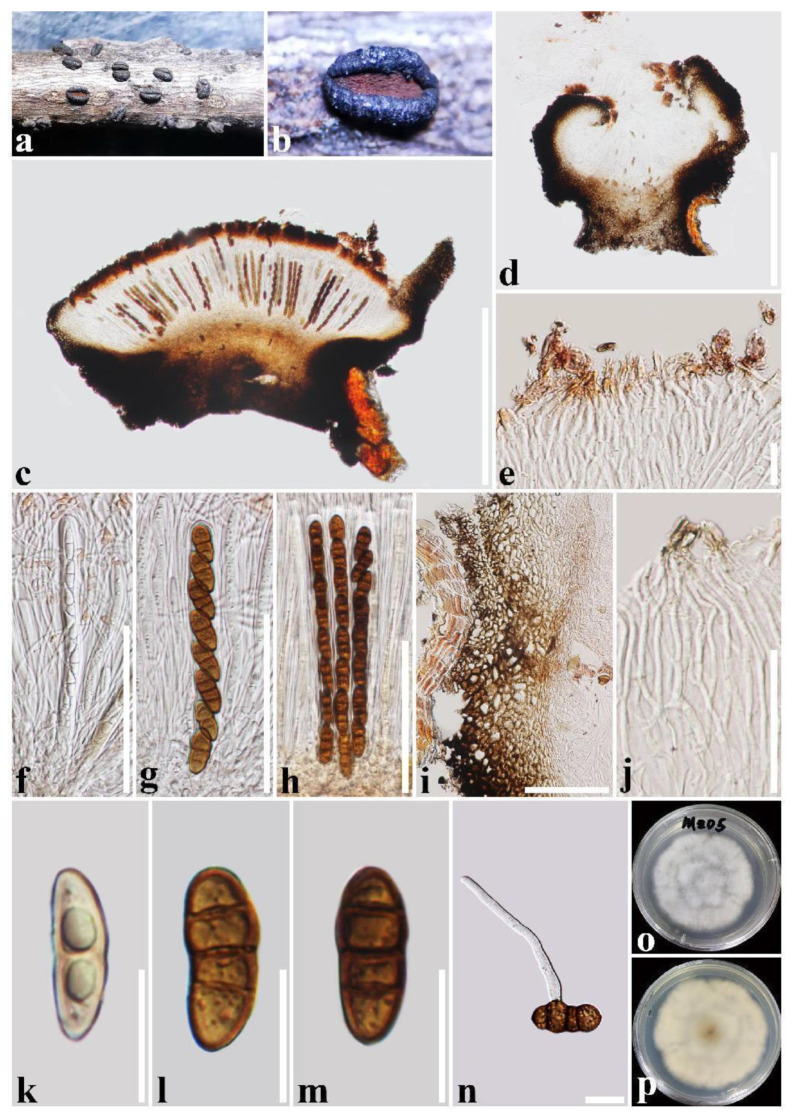
***Rhytidhysteron mengziense***(HKAS 122699, **holotype**). (**a**,**b**) Appearance of hysterothecia on the host; (**c**,**d**) Vertical section through hysterothecium; (**e**) Epithecium mounted in water; (**f**–**h**) Asci; (**i**) Exciple; (**j**) Pseudoparaphyses; (**k**–**m**) Ascospores; (**n**) A germinating ascospore; (**o**,**p**) Colony on PDA medium (after one week). Scale bars: (**c**,**d**) = 500 μm; (**e**,**k**–**n**) = 20 μm; (**f**–**i**) = 100 μm; (**j**) = 50 μm.

***Rhytidhysteron mengziense*** T.Y. Du and Tibpromma sp. nov. (Figure 4)

MycoBank number: MB 846001. Facesoffungi number: FoF 12959

Etymology: Named after the region Mengzi where the type specimen was collected.

Holotype: HKAS 122699

*Saprobic* on decaying twigs of *Crataegus scabrifolia* (Rosaceae). Sexual morph: *Ascomata*: 1000–1600 μm long × 800–1000 μm wide × 400–700 μm high (x = 1400 × 910 × 640 μm, n = 5), hysterothecial, solitary to aggregated, mostly solitary, semi-immersed to superficial, navicular, black, apothecioid, smooth, perpendicular striae, elongate and depressed, compressed at apex, opening through a longitudinal slit, reddish brown at the center. *Exciple*: 60–135 μm wide, composed of outer layer brown to black, thick-walled cells of *textura angularis*, and inner layer light brown, thin-walled cells of *textura prismatica*. *Hamathecium*: 1–2.5 μm wide, dense, hyaline, septate, branched, cellular pseudoparaphyses, forming a reddish brown to brown epithecium above asci when mounted in water, becoming purple epithecium above the asci when mounted in 10% KOH, and turns hyaline after 30 s, while appendages turn dark brown. *Asci*: 150–176(–182) μm × 10–14(–16.3) μm (x = 164.5 × 13 μm, n = 20), 8-spored, bitunicate, cylindrical, with short pedicel, rounded at the apex, with an ocular chamber, J- apical ring, always fused with hamathecium. *Ascospores*: (22.5–)24.5–27.5(–29) μm × 10.5–12.5 μm (x = 27 × 12 μm, n = 30), slightly overlapping, uni-seriate, slightly overlapping, hyaline, 1-septate when immature, becoming reddish brown to brown, (1–)3-septate when mature, ellipsoidal to fusoid, straight or curved, rounded to slightly pointed at both ends, guttulate, rough-walled, without the mucilaginous sheath. Asexual morph: Undetermined.

Culture characteristics: Ascospores germinated on PDA within 24 h and germ tubes produced from one or both ends. Colonies on PDA reached a 6 cm diameter after one week at 28 °C. The colony is flossy, velvety, circular, slightly raised, with an undulated edge, white aerial hyphae on the forward and cream white in reverse. 

Material examined: China, Yunnan Province, Honghe Prefecture, Mengzi City, on a decaying piece of wood of *Crataegus scabrifolia* (Rosaceae), 21 May 2020, S. Tibpromma, MZD5 (holotype, HKAS 122699, ex-type living culture, KUMCC 21-0490 = KUMCC 21-0491).

Notes: In phylogenetic analyses, *Rhytidhysteron mengziense* was well separated from *R. camporesii* with low statistical support. However, *R. mengziense* is distinct from *R. camporesii* in having exciple cells of *textura angularis* to *prismatica*, and rough-walled ascospores, while *R. camporesii* has exciple cells of *textura globulosa* to *angularis*, and smooth-walled of ascospores [49]. In addition, the ascomata and ascospore size of *R. mengziense* are larger than those of *R. camporesii* (ascomata: 1400 × 640 μm vs. 1002.4 × 570.1 µm, ascospores: 27 × 12 μm vs. 26.1 × 10.4 µm) [49]. Moreover, according to the comparison results of different gene fragments, *R. mengziense* is different from *R. camporesii* in ITS gene (10/651 bp, 1.54%, without gaps). Therefore, in this study, *R. mengziense* is introduced as a new species. 

***Rhytidhysteron neorufulum*** Thambug. & K.D. Hyde, Cryptog. Mycol. 37(1): 110 (2016) (Figure S5)

MycoBank number: MB 551865. Facesoffungi number: FoF 01840

Description: See Thambugala et al. [16], Supplementary Notes 5

Distribution: China (this study), Mexico [55], Thailand [16,18].

Host: *Bursera* sp. [55], *Elaeagnus sarmentosa* (this study), *Hevea brasiliensis* [16], *Tectona grandis* [18].

Material examined: China, Yunnan Province, Xishuangbanna Prefecture, Mengla County, Xishuangbanna Tropical Botanical Garden, 101°15′45″ E, 21°55′50″ N, on decaying wood of *Elaeagnus sarmentosa* Rehd. (Elaeagnaceae), 24 November 2020, T.Y. Du, BND49 (HKAS 122691, living culture, KUMCC 21-0480).

Notes: *Rhytidhysteron neorufulum* was introduced by Thambugala et al. [16] based on both morphology and phylogenetic analyses. According to phylogenetic analyses based on combined multi-gene (LSU, ITS, SSU, and *TEF*), our collection grouped together with *R. neorufulum* (MFLUCC 13-0216, GKM 361A, HUEFS 192194, MFLUCC 12-0528, CBS 306.38, MFLUCC 12-0011, MFLUCC 12-0567, MFLUCC 12-0569, EB 0381, MFLUCC 21-0035). In addition, our collection shows similar morphological characteristics to *R. neorufulum*, having solitary to aggregated, superficial, non-striated hysterothecia, septate pseudoparaphyses, 8-spored, cylindrical, short pedicellate asci, and uni-seriate, ellipsoidal to fusiform, ascospores, brown when mature [16,18]. This species was previously only recorded in Thailand and Mexico. Therefore, we report our collection as new geographical and host record of *R. neorufulum* from the decaying wood of *Elaeagnus sarmentosa* (Elaeagnaceae) in the Yunnan Province of China. This is the first record of *R. neorufulum* on *Elaeagnus sarmentosa*. 

***Rhytidhysteron tectonae*** Doilom & K.D. Hyde, Fungal Diversity. 82: 107–182 (2017) (Figure S6)

MycoBank number: MB 551964. Facesoffungi number: FoF 01849

Description: See Doilom et al. [53], Supplementary Notes 6

Distribution: China (this study), Thailand [18,53].

Host: *Betula* sp., an unidentified member of the Fabaceae [18], *Magnolia delavayi* (this study), and *Tectona grandis* [53].

Material examined: China, Yunnan Province, Xishuangbanna Prefecture, Mengla County, Xishuangbanna Tropical Botanical Garden, 101°15′25″ E, 21°55′37″ N, on decaying wood of *Magnolia delavayi* Franch. (Magnoliaceae), 24 November 2020, T.Y. Du, BND33 (HKAS 122692, living culture, KUMCC 21-0479).

Notes: *Rhytidhysteron tectonae* was introduced by Doilom et al. [53] based on both morphology and phylogenetic analyses. According to phylogenetic analyses based on combined multi-gene (LSU, ITS, SSU, and *TEF*), our collection grouped together with *R. tectonae* (MFLUCC 13-0710, MFLUCC 21-0034, and MFLUCC 21-0037). In addition, our collection shows similar morphological characteristics to *R. tectonae*, having solitary to aggregated, semi-immersed to superficial, non-striated, yellow at the center hysterothecia, septate, branched pseudoparaphyses, 8-spored, cylindrical, short pedicellate asci, and uni-seriate, slightly overlapping, 1–3-septate ascospores, dark brown when mature [18,53]. This species was previously only recorded in Thailand. Therefore, we report our collection as a new geographical and host record of *R. tectonae* from decaying wood of *Magnolia delavayi* (Magnoliaceae) in the Yunnan Province of China. This is the first record of *R. tectonae* on *Magnolia delavayi*. 

***Rhytidhysteron thailandicum*** Thambug. & K.D. Hyde, Cryptog. Mycol. 37(1): 110 (2016) (Figure S7)

MycoBank number: MB 551866. Facesoffungi number: FoF 01841

Description: See Thambugala et al. [16], Supplementary Notes 7

Distribution: China [6] (this study), Mexico [55], Thailand [16,54].

Host: *Acacia* sp. [55], *Afzelia xylocarpa* [54], *Aquilaria sinensis* (this study), *Morus australis* [6], and unidentified wood [16].

Material examined: China, Yunnan Province, Xishuangbanna Prefecture, Menghai County, agarwood plantation, on decaying wood of *Aquilaria sinensis* (Lour.) Spreng. (Thymelaeaceae), 15 September 2021, T.Y. Du, YNA62 (HKAS 122689, living culture, KUMCC 21-0493).

Notes: *Rhytidhysteron thailandicum* was introduced by Thambugala et al. [16] based on both morphology and phylogenetic analyses. According to phylogenetic analyses based on combined multi-gene (LSU, ITS, SSU, and *TEF*), our collection grouped together with *R. thailandicum* (MFLUCC 14-0503, MFLUCC 12-0530, MFLU17-0788, and MFLUCC 13-0051). In addition, our collection shows similar morphological characteristics to *R. thailandicum*, having solitary to aggregated, globose to subglobose, striated hysterothecia, exciple cells of *textura angularis*, septate, branched pseudoparaphyses, cylindrical, short pedicellate asci, and uni-seriate, brown ascospores [6,16,54]. There is a small difference between our collection and *R. thailandicum* in that the surface of the ascomata of our collection is covered with green. However, in the phylogenetic tree, our collection (KUMCC 21-0493) grouped with the four strains of *R. thailandicum*. Therefore, we report our collection as a new host record of *R. thailandicum* from decaying wood of *Aquilaria sinensis* (Thymelaeaceae) in the Yunnan Province of China. This is the first record of *R. thailandicum* on *Aquilaria sinensis*. 

**Figure 5:**
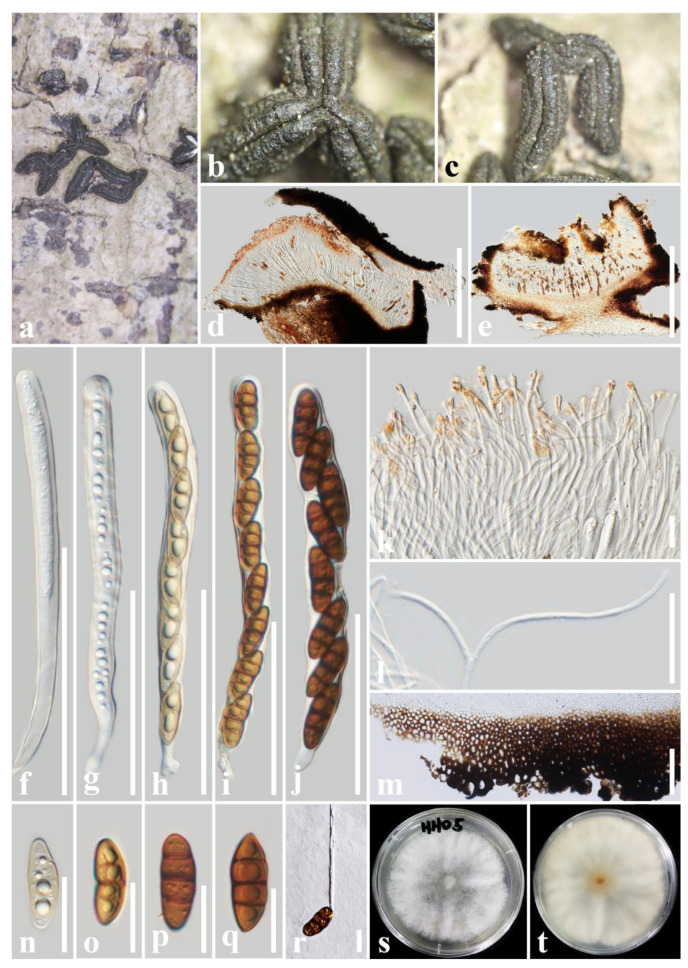
***Rhytidhysteron yunnanense*** (HKAS 122696, **holotype**). (**a**–**c**) Appearance of hysterothecia on the host; (**d**,**e**) Vertical section through hysterothecium; (**f**–**j**) Asci; (**k**) Epithecium mounted in water; (**l**) Pseudoparaphyses; (**m**) Exciple; (**n**–**q**) Ascospores; (**r**) A germinating ascospore; (**s**,**t**) Colony on PDA medium (after one week); Scale bars: (**d**,**e**) = 500 μm; (**f**–**j**) = 100 μm; (**k**,**l**,**n**–**r**) = 20 μm; (**m**) = 50 μm.

***Rhytidhysteron yunnanense*** T.Y. Du and Tibpromma, sp. nov. (Figure 5)

MycoBank number: MB 846002. Facesoffungi number: FoF 12960

Etymology: Named after the region Yunnan where the type specimen was collected.

Holotype: HKAS 122696

*Saprobic* on decaying wood of *Rhus chinensis* (Anacardiaceae). Sexual morph: *Ascomata*: 1900–3000 μm long × 400–800 μm wide × 300–600 μm high (x = 2510 × 625 × 455 μm, n = 5), hysterothecial, solitary to aggregated, mostly aggregated, semi-immersed, navicular to irregular, black, apothecioid, rough, each hysterothecia has two parallel striae parallel to the longitudinal slit, and slight perpendicular striae, elongate and depressed, compressed at apex, longitudinal slit, no opening. *Exciple*: 60–180 μm wide, composed of dark brown, thick-walled cells of *textura globulosa*, outer layer brown to dark brown, inner layer pale brown to hyaline. *Hamathecium*: 1–2.5 μm wide, dense, hyaline, septate, branched, cellular pseudoparaphyses, forming a yellow epithecium above asci when mounted in water, and becoming hyaline epithecium above the asci when mounted in 10% KOH, while appendages turn dark. *Asci*: (205–)215–250(–265) μm × 12–16(–17) μm (x = 230 × 14 μm, n = 20), 8-spored, bitunicate, cylindrical, short with club-like perdicel, rounded at the apex, with an ocular chamber, J- apical ring. *Ascospores*: 28.5–36 μm × 11–14.5 μm (x = 32.5 × 13 μm, n = 30), uni-seriate when mature, hyaline, 1-septate when immature, becoming reddish brown to brown, 3-septate when mature, ellipsoidal to fusoid, straight or curved, rounded to slightly pointed at both ends, guttulate, without the mucilaginous sheath. Asexual morph: Undetermined.

Culture characteristics: Ascospores germinated on PDA within 24 h and germ tubes produced from one or both ends. Colonies on PDA reached a 6 cm diameter after two weeks at 28 °C. The colony is velvety, circular, slightly raised, with a filiform edge, white on the forward and white in reverse. 

Material examined: China, Yunnan Province, Honghe Prefecture, Honghe County, 102°14′24″ E, 23°25′30″ N, on a decaying wood piece of *Rhus chinensis* Mill. (Anacardiaceae), 8 December 2020, T.Y. Du, HHD5 (holotype, HKAS 122696, ex-type living culture, KUMCC 21-048 = KUMCC 21-0486).

Notes: In phylogenetic analyses, *Rhytidhysteron yunnanense* was well separated from *R. mesophilum* with good statistical support (90% ML/0.92 PP). In morphology, *R. yunnanense* is distinct from *R. mesophilum*, having navicular to irregular ascomata, each hysterothecia has two parallel striae parallel to the longitudinal slit, and slight perpendicular striae, and a longitudinal slit with no opening, while *R. mesophilum* has boat-shaped ascomata, with perpendicular striae with a perpendicular to longitudinal slit, and a longitudinal slit opening [24]. In addition, the asci and ascospore size of *R. yunnanense* are smaller than those of *R. mesophilum* (asci: 230 × 14 μm vs. 267–282 × 15.5–16 µm, ascospores: 32.5 × 13 μm vs. 44.2 × 13.6 μm) [24]. Moreover, according to the comparison results of different gene fragments, *R. yunnanense* is different from *R. mesophilum* in the ITS (28/651 bp, 4.30%, without gaps) gene. Therefore, in this study, *R. yunnanense* is introduced as a new species. 

Moreover, in the previous study of the genus *Rhytidhysteron*, ascomata have a transverse-striae, perpendicular to the longitudinal slit, and this study is the first to find parallel striae that are parallel to the longitudinal slit. 

## 4. Discussion

Based on the morphological study and phylogenetic analyses, four new species and seven new records of *Rhytidhysteron* are introduced in this paper. *Rhytidhysteron bannaense* sp. nov., *R. coffeae* sp. nov., *R. mengziense* sp. nov., and *R. yunnanense* sp. nov. are proposed as new to science based on their unique morphological characteristics and moderate to good statistical support. Seven collections of *R. bruguierae*, *R. camporesii*, *R. hongheense*, *R. magnoliae*, *R. neorufulum*, *R. tectonae*, and *R. thailandicum* are identified as new records because of their identical morphological characteristics with the type species of the same species and high statistical support.

In this study, we found that the pseudoparaphyses of all 11 species are branched and septate. Interestingly, the epithecia of ten species all turn purple in 10% KOH, but the purple color of the epithecium fades and becomes hyaline in a short period of time (5–30 s). On the contrary, the epithecium of *R. yunnanense* sp. nov. (HKAS 122696) turns hyaline in 10% KOH, and the appendages become dark. In addition, *R. yunnanense* is unique in *Rhytidhysteron* because it has both parallel and perpendicular striae, relative to the longitudinal slit. This is also the first discovery of parallel striae in this genus, while other species of *Rhytidhysteron* have perpendicular striae or are non-striated (Table 3). The presence or absence of striae on the margin of ascomata is one of the important characteristics of this genus and is used to identify different species [16]. 

The results of the phylogenetic tree generated in this study are consistent with those reported by Ren et al. [18], and *R. erioi* Ekanayaka & K.D. Hyde is grouped as a sister to *R. bruguierae*. Therefore, to find out the correct taxonomic placement of *R. erioi*, it is necessary to recollect more samples and confirm their placement. Boehm et al. [13] suggested that *R. opuntiae* (J.G. Br.) M.E. Barr should be removed from *Rhytidhysteron* based on morphological and molecular data. Subsequently, Almeida et al. [15] suggested that *R. opuntiae* should be accommodated by a new genus in future studies. The main reason is that *R. opuntiae* grouped with *Hysterodifractum partisporum* D.A.C. Almeida, Gusmão & A.N. Mill. [15,56], but morpho-molecular differences exist between *Hysterodifractum* D.A.C. Almeida, Gusmão & A.N. Mill. and *R. opuntiae* [12]. This study agrees with Boehm et al. [13] and Almeida et al. [15] in that *R. opuntiae* should be included in a new genus, due to the fact that morpho-molecular data of *R. opuntiae* are different from those of *Rhytidhysteron* and *Hysterodifractum*. Therefore, more studies on *R. opuntiae* are needed.

*Rhytidhysteron neorufulum* and *R. rufulum* are the most common and most reported species in this genus [51]. *Rhytidhysteron rufulum* is considered a complex species based on studies on molecular and chemical data [16,22,27,51,57]. Thambugala et al. [16] indicated that some fungal specimens were incorrectly classified as *R. rufulum*, which needs to be reviewed exhaustively, as they might represent new species [51]. This action is meaningful, and can clarify the taxonomic placement of unclear species and enrich the diversity of *Rhytidhysteron*. Unfortunately, in this study, no strains with very similar morpho-molecular data to *R. rufulum* were found.

In previous studies, six species were reported in China. These are *R. camporesii* [49], *R. hongheense* [25], *R. magnoliae* [6], *R. rufulum* [15], *R. thailandicum* [6], and *R. xiaokongense* [18]. This study provides seven additional species from China—*R. bannaense* sp. nov., *R. bruguierae*, *R. coffeae* sp. nov., *R. mengziense* sp. nov., *R. neorufulum*, *R. tectonae*, and *R. yunnanense* sp. nov. With these additions, the number of species of *Rhytidhysteron* in China increases from six to thirteen. At the same time, the four new species added in this study expand the species of *Rhytidhysteron* from thirty-three to thirty-seven, and the known hosts for *Rhytidhysteron* expand from 52 to 62 records. However, only 22 species of *Rhytidhysteron* have sequence data (including this study), so more research needs to be carried out, and more samples need to be collected, isolated, and sequenced. This study also summarizes the morphological characteristics, hosts, and countries of the species of this genus for the first time (Table 3), which provides references for future research on *Rhytidhysteron*.

## Figures and Tables

**Table 1:** Taxa name, strain numbers, and GenBank accession numbers included in the phylogenetic analyses carried out in the present study.

Taxa Name	Strain Number	GenBank Accession Numbers				References
		LSU	ITS	SSU	*TEF*	
*Gloniopsis calami*	MFLUCC 15-0739	NG059715	KX669036	KX669034	KX671965	[46]
*G. praelonga*	CBS 112415	FJ161173	—	FJ161134	FJ161090	[13]
***Rhytidhysteron bannaense***	**KUMCC 21-0482 ^T^**	**OP526408**	**OP526398**	**OP526395**	**OP572199**	**This study**
***R. bannaense***	**KUMCC 21-0483**	**OP526409**	**OP526399**	**OP526396**	**OP572200**	**This study**
***R. bruguierae***	**KUMCC 21-0484**	**OP442285**	**OP494090**	**OP482277**	**OP572207**	**This study**
*R. bruguierae*	MFLU 18-0571 ^T^	MN017833	—	MN017901	MN077056	[47]
*R. bruguierae*	MFLUCC 17-1515	MN632452	MN632457	MN632463	MN635661	[48]
*R. bruguierae*	MFLUCC 17-1511	MN632454	MN632459	MN632465	—	[48]
*R. bruguierae*	MFLUCC 17-1502	MN632453	MN632458	MN632464	MN635662	[48]
*R. bruguierae*	MFLUCC 17-1509	MN632455	MN632460	MN632466	—	[48]
***R. camporesii***	**KUMCC 21-0488**	**OP482286**	**OP494091**	**OP482278**	**OP572208**	**This study**
*R. camporesii*	KUN-HKAS 104277 ^T^	MN429072	MN429069	—	MN442087	[49]
*R. chromolaenae*	MFLUCC 17-1516 ^T^	MN632456	MN632461	MN632467	MN635663	[48]
***R. coffeae***	**KUMCC 21-0489 ^T^**	**OP526406**	**OP605963**	**OP526412**	**OP572201**	**This study**
***R. coffeae***	**KUMCC 21-0492**	**OP526407**	**OP605964**	**OP526413**	**OP572202**	**This study**
*R. cozumelense*	A. Cobos-Villagrán 951	MW939459	MZ056797	—	MZ457338	[24]
*R. cozumelense*	T. Raymundo 7321	MW939460	MZ056798	—	MZ457339	[24]
*R. erioi*	MFLU 16-0584 ^T^	MN429071	MN429068	—	MN442086	[49]
*R. esperanzae*	T. Raymundo 6579	MZ477203	MZ056795	—	MZ457336	[24]
*R. esperanzae*	R. Valenzuela 17206	MZ477204	MZ056796	—	MZ457337	[24]
***R. hongheense***	**KUMCC 21-0487**	**OP482287**	**OP494092**	**OP482279**	**OP572209**	**This study**
*R. hongheense*	KUMCC 20-0222	MW264193	MW264214	MW264223	MW256815	[25]
*R. hongheense*	HKAS112348	MW541820	MW541824	MW541831	MW556132	[25]
*R. hongheense*	HKAS112349	MW541821	MW541825	MW541832	MW556133	[25]
*R. hysterinum*	EB 0351	GU397350	—	—	GU397340	[13]
*R. hysterinum*	CBS 316.71	MH871912	MH860141	—	—	[50]
***R. magnoliae***	**KUMCC 21-0478**	**OP482288**	**OP494093**	**OP482280**	**OP572210**	**This study**
*R. magnoliae*	MFLUCC 18-0719 ^T^	MN989384	MN989383	MN989382	MN997309	[6]
*R. mangrovei*	MFLU 18-1894 ^T^	MK357777	MK425188	—	MK450030	[12]
***R. mengziense***	**KUMCC 21-0490 ^T^**	**OP526396**	**OP526402**	**OP526414**	**OP572203**	**This study**
***R. mengziense***	**KUMCC 21-0491**	**OP526397**	**OP526403**	**OP526415**	**OP572204**	**This study**
*R. mesophilum*	A. Trejo 74	MW939461	MZ056799	—	MZ457340	[24]
*R. mesophilum*	A. Cobos-Villagrán 1800	MW939462	MZ056800	—	MZ457341	[24]
*R. mexicanum*	RV17107.1 ^T^	MT626028	MT626026	—	—	[51]
*R. mexicanum*	RV17107.2	MT626029	MT626027	—	—	[51]
***R. neorufulum***	**KUMCC 21-0480**	**OP482290**	**OP494095**	**OP482282**	**OP572212**	**This study**
*R. neorufulum*	MFLUCC 21-0035	MZ346015	MZ346020	MZ346025	MZ356249	[18]
*R. neorufulum*	MFLUCC 13-0216 ^T^	KU377566	KU377561	KU377571	KU510400	[16]
*R. neorufulum*	GKM 361A	GQ221893	—	GU296192	—	[13]
*R. neorufulum*	HUEFS 192194	KF914915	—	—	—	[15]
*R. neorufulum*	MFLUCC 12-0528	KJ418117	KJ418118	KJ418119	—	[16]
*R. neorufulum*	CBS 306.38	FJ469672	—	GU296191	GU349031	[15]
*R. neorufulum*	MFLUCC 12-0011	KJ418109	KJ206287	KJ418110	—	[16]
*R. neorufulum*	MFLUCC 12-0567	KJ526126	KJ546124	KJ546129	—	[16]
*R. neorufulum*	MFLUCC 12-0569	KJ526128	KJ546126	KJ546131	—	[16]
*R. neorufulum*	EB 0381	GU397351	—	GU397366	—	[13]
*R. opuntiae*	GKM 1190	GQ221892	—	—	GU397341	[52]
*R. rufulum*	MFLUCC 14-0577 ^T^	KU377565	KU377560	KU377570	KU510399	[16]
*R. rufulum*	EB 0384	GU397354	—	GU397368	—	[13]
*R. rufulum*	EB 0382	GU397352	—	—	—	[13]
*R. rufulum*	EB 0383	GU397353	—	GU397367	—	[13]
*R. rufulum*	MFLUCC 12-0013	KJ418111	KJ418112	KJ418113	—	[6]
***R. tectonae***	**KUMCC 21-0479**	**OP482291**	**OP494096**	**OP482283**	**OP572213**	**This study**
*R. tectonae*	MFLUCC 21-0037	MZ346013	MZ346018	MZ346023	MZ356247	[18]
*R. tectonae*	MFLUCC 21-0034	MZ346014	MZ346019	MZ346024	MZ356248	[18]
*R. tectonae*	MFLUCC 13-0710 ^T^	KU764698	KU144936	KU712457	KU872760	[53]
***R. thailandicum***	**KUMCC 21-0493**	**OP482292**	**OP494097**	**OP482284**	**OP572214**	**This study**
*R. thailandicum*	MFLUCC 14-0503 ^T^	KU377564	KU377559	KU377569	KU497490	[16]
*R. thailandicum*	MFLUCC 12-0530	KJ526125	KJ546123	KJ546128	—	[16]
*R. thailandicum*	MFLU17-0788	MT093472	MT093733	MT093495	—	[6]
*R. thailandicum*	MFLUCC 13-0051	MN509434	MN509433	—	MN509435	[54]
*R. xiaokongense*	KUMCC 20-0158	MZ346011	MZ346016	MZ346021	MZ356245	[18]
*R. xiaokongense*	KUMCC 20-0160 ^T^	MZ346012	MZ346017	MZ346022	MZ356246	[18]
***R. yunnanense***	**KUMCC 21-0485 ^T^**	**OP526404**	**OP526410**	**OP526400**	**OP572205**	**This study**
***R. yunnanense***	**KUMCC 21-0486**	**OP526405**	**OP526411**	**OP526401**	**OP572206**	**This study**

**Remarks:** The newly generated sequences are indicated in bold, the superscript ^T^ indicates ex-type, and “—” indicates information unavailable.

**Table 2:** Comparison of LSU, ITS, LSU, and *TEF* gene fragments of *R. bannaense* and four strains of *R. thailandicum* (without gaps).

Closest Known Species	Strain Number	LSU Gene	ITS Gene	SSU Gene	*TEF* Gene	References
*R. thailandicum*	MFLUCC 14-0503 ^T^	0.00%	1.79% (9/503 bp)	0.00%	2.26% (21/929 bp)	[16]
	MFLUCC 12-0530	0.00%	1.89% (10/527 bp)	0.00%	—	[16]
	MFLU 17-0788	0.00%	1.42% (7/494 bp)	0.00%	—	[6]
	MFLUCC 13-0051	0.00%	1.81% (9/496 bp)	—	2.28% (21/923 bp)	[54]

**Remarks:** The “—” represents unavailable data for this gene, superscript ^T^ indicates the ex-type.

**Table 3:** Morphological characteristics, hosts, and location information of species of *Rhytidhysterons*. Host and location information of taxa are from the following references and Farr and Rossman [26].

Species	Ascomata/ Conidiomata	Exciples/Conidiomata Wall	Hamathecium/ Paraphysoids	Asci/ Conidiogenous Cells	Ascospores/ Conidia	Sequences	Hosts	Countries	References
Holomorph									
*R. hysterinum*	Sexual: ascomata 1000–3000 µm long × 500 µm wide × 500–1000 µm high, smooth-striated, erumpent, solitary or aggregated, sessile, deep longitudinal slit extending the entire length of the ascoma and with irregularly spaced, pseudoepithecium, orange or black when fresh and when dry	Exciples tightly compact, hyaline to light brown, becoming red in Melzer’s reagent, cells toward the interior less heavily pigmented	Paraphyses exceeding the ascus by ca. 25 µm, branching dichotomously just below the tip, tip cells globose to clavate, disintegrating and embedded in an amorphous substance to form the pseudoepithecium, becoming blue-green in Melzer’s reagent	Asci 185–220 µm × 15–17 µm, 4–8-spored, cylindrical, pedicellate	Ascospores 21–32 µm × 8–12 µm, 1-septate, septum median, fusiform with rounded to acute ends, slightly constricted at the septum, brown and translucent to nearly black, and opaque with septum obscured	ITS, LSU, *TEF*	*Buxus* spp., *Diospyros* sp., *Ilex* sp., *Prosopis* sp.	Australia, France, India, USA	[13,21]
	**Asexual (*Aposphaeria*-like)**: pycnidia produced abundantly in the aerialmycelium and on the surface of the agar on both MEA and PDA, non-stromatic, globose with a short papilla, 250–330 µm high × 220–250 µm wide, black	—	—	Phialides forming in a single layer over the entire inner surface of the pycnidial wall, hyaline, ampulliform to cylindrical, 7–8 µm long, × 2 µm wide basally, and 1.5 µm wide at the opening	Conidia globose, 2–3.5 µm diam., smooth, held in hyaline slime at the pycnidial opening				
	**Asexual (*Diplodia*-like)**: pycnidia form on MEA within one month, immersed, non-stromatic, subglobose, 350 µm high × 400 µm wide, non-papillate, black, smooth	Pycnidial wall 25–35 µm wide, consisting of pseudoparenchymatous cells, 5–7 µm × 3–4 µm, thin-walled, brown	Paraphyses arisingfrom among conidiogenous cells, up to 50 µm long × 3 µm wide, septate, unbranched, with rounded ends, hyaline	Conidiogenous cells forming in a single layer over the entire inner surface of the pycnidial wall, barely distinguishable from cells of the wall; consisting of a basal cell 6–7 µm across, and a 5–10 µm long elongation	Conidia arising holoblastically from the tip of the conidiogenous cell; at first hyaline and unicellular, becoming dark brown, opaque, minutely punctate, and 1-septate with a pore in the middle of the septum, oblong, with a truncate, non-cicatrized base, 22–26 µm × 9–11 µm				
*R. rufulum*	**Sexual**: ascomata 900–2350 µm long × 1134–1450 µm wide × 461–820 µm high, superficial, rough-striated, black or red at the center	Exciples 75–228 µm wide, two layers. Outer layer dark brown to black, cells of *textura angularis* or *textura globosa*. Inner layer hyaline cells of *textura angularis* to *textura prismatica*	Septate, branched pseudoparaphyses	Asci 150–250 µm × 11–16 µm, 8-spored	Ascospores 28–36 µm × 9–13 µm, 1–3-septate, reddish brown to brown when mature	ITS, LSU, SSU, *TEF*	*Abrus precatorius*, *Abrus pulchellus*, *Acacia auriculiformis*, *Acacia cochliacantha*, *Acacia farnesiana*, * Acacia macracantha*, *Acacia* spp., *Adhatoda vasica*, *Albizia lebbeck*, *Albizia odoratissima*, *Alphitonia excelsa*, *Annona muricata*, *Bignonia unguis*, *Bougainvillea glabra*, *Capparis sepiaria*, *Casuarina* sp., *Celtis pallida*, *Citrus aurantifolia*, *Citrus aurantium*, *Codiaeum variegatum*, *Euterpe oleracea*, *Grevillea robusta*, *Guaiacum officinale*, *Helietta parvifolia*, *Juniperus lucayana*, *Nothofagus* sp., *Pisonia aculeata*, *Pithecellobium dulce*, *Prosopis juliflora*, *Torresia cearensis*	Argentina, Australia, Brazil, China, Cook Islands, Costa Rica, Cuba, Dominica, France, Ghana, India, Jamaica, Japan, Kenya, Malaysia, Mexico, Micronesia, New Guinea, New Zealand, Philippine, Puerto, Rico, Spain, Tanzania, Thailand, Tonga, United States, Venezuela, West Indies	[15,16,21,55,58,59,60,61,62,63,64,65,66,67,68,69,70]
	**Asexual (*Aposphaeria*-like)**: pycnidia form abundantly in aerial mycelium, often associated with small tufts of red-brown hyphae, non-stromatic, globose to oblong, 100–150 µm high × 70–150 µm wide, non-papillate, black	—	—	Phialides forming in a single layer over the entire inner surface of the pycnidial wall, hyaline, ampulliform to cylindrical, 4.5–9.0 µm long × 1.5–3.0 µm diam. basally, and 1.5 µm wide at the opening	Conidia globose to elliptic, 2–3 µm diam. or 3.0 µm × 2.5 µm, smooth, held in a drop of hyaline slime at the pycnidial opening				
	Asexual (*Diplodia*-like): pycnidia abundant to rare, immersed, non-stromatic, subglobose, 460 µm high × 400 µm wide, papillate or non-papillate, or seated on the surface of the agar, pyriform, 270 µm high × 130–180 µm wide and with a papilla 130–180 µm long × 70 µm wide, black, smooth	Pycnidial wall 45 µm wide, consisting of pseudoparenchymatous cells 8–20 µm × 8–10 µm, thin-walled, brown	Paraphyses arising from among conidiogenous cells, up to 50 µm long × 3 µm wide, septate, unbranched, with rounded ends, hyaline	Conidiogenous cells forming in a single layer over the entire inner surface of the pycnidial wall, consisting of a hyaline, globose cell 4–5 µm in diam. basally, and with a 5 µm long elongation	Conidia arising holoblastically from the tip of the elongation of the conidiogenous cell, at first hyaline and unicellular, becoming dark brown to opaque and 1-septate with a pore in the middle of the septum following discharge, oblong with a truncate, non-cicatrized base, 19.5–23.5 (–29.5) µm × (6.5–) 8–10 (–12) µm, smooth				
*R. thailandicum*	**Sexual**: ascomata 700–1200 µm long× 530–750 µm wide × 360–640 µm high, semi-immersed to superficial, rough without striations	Exciples 72–130 μm wide, brown to dark brown, thick-walled cells of *textura angularis*, becoming hyaline towards the inner layers and base	Septate, branched pseudoparaphyses, forming a yellow epithecium above asci when mounted in water	Asci 135–160 µm × 10.5–15 µm, (3–)6–8-spored	Ascospores 20–31 µm × 7.5–12 µm, (1–)3-septate, yellowish to brown when mature	ITS, LSU, SSU, *TEF*	*Acacia* sp., *Aquilaria sinensis*, Morus australis	China, Mexico, Thailand	[6,16,55], this study
	Asexual (*Aposphaeria*-like): conidiomata 70–108 µm long × 63–110 µm wide, superficial on PDA, globose, black, appearing in a mycelium mass	Conidiomata wall thin, arranged in *textura angularis*	—	Conidiophores reduced to conidiogenous cells. Conidiogenous cells 5.9 µm × 3 µm, cylindrical to subcylindrical, truncate apex, short, smooth, hyaline	Conidia 2.9 µm × 2.2 µm, globose to subglobose, hyaline, smooth				
**Asexual morph**									
*R. xiaokongense*	**Asexual (*Diplodia*-like):** conidiomata 448–464 µm long × 324–422 µm wide, solitary, scattered, semi-immersed in the host, black, unilocular, subglobose to ampulliform. Ostioles 178–227 µm long × 166–234 µm wide, central, short papillate	Conidiomata wall 30–40 µm thick, 4–6 layers, reddish brown to dark brown cells of *textura angularis*	—	Conidiogenous cells 5–8 µm × 3–6 µm, subglobose or ellipsoidal, hyaline, smooth, discrete, producing a single conidium at the apex	Conidia 20–25 µm × 8–10 µm, 1-septate and brown to dark brown at maturity, oblong to ellipsoidal, straight to slightly curved, with granular appearance	ITS, LSU, SSU, *TEF*	*Prunus* sp.	China	[18]
**Sexual morph**									
*R. bannaense*	1350 µm long × 750 µm wide × 670 µm high, rough, solitary to aggregated, semi-immersed to superficial, perpendicular striae, green at the center	40–150 µm wide, composed of dark brown, thick-walled cells of *textura angularis*, outer layer brown to dark brown, inner layer pale brown to hyaline	Septate, branched, cellular pseudoparaphyses, forming an orange epithecium above asci when mounted in water	166 µm × 14 µm, 8-spored, J- apical ring	25 µm × 11.5 µm, 3-septate, brown to dark brown when mature	ITS, LSU, SSU, *TEF*	*Buddleja officinalis*	China	This study
*R. beccarianum*	1000 µm long, erumpent, solitary, dark brown	—	—	60 µm × 6 µm, 8-spored	12–15 µm × 5–6 µm, 3-septate, constriction at the septa	—	—	Sri Lanka	[71]
*R. brasiliense*	Erumpent to nearly superficial	—	Septate, branched pseudoparaphyses	230–250 µm × 20–30 µm, 8-spored	40–45 µm × 15–20 µm, 3-septate	—	On rotten branches	Brazil, Thailand	[16,19]
*R. bruguierae*	400–950 µm long × 548–570 µm wide × 410–520 µm high, superficial, striated	148–162 µm wide, dark brown to black, thick-walled cells of *textura angularis*	Septate, branched pseudoparaphyses, forming a red epithecium above asci when mounted in water	128–148 µm × 10–14 µm, 6–8-spored, J- apical ring	14–26 µm × 6.2–9 µm, 1–3-septate, yellowish-brown to reddish brown when mature	ITS, LSU, SSU, *TEF*	*Alnus nepalensis, Bruguiera* sp., Chromolaena odorata	China, Thailand	[47,48], this study
*R. camporesii*	800–1100 µm long × 500–650 µm high, erumpent, slightly dentate	Ectal excipulum 65–95 µm wide, blackish cells of *textura globulosa* to *angularis*. Medullary excipulum 19–22 µm wide, thin-walled, hyaline to brown cells of *textura porrecta*	Paraphyses septate, branched at the base, forming an orange-red epithecium above asci when mounted in water	165–175 µm × 13–15 µm, 8-spored, J- apical ring	25–28 µm × 9–11 µm, 3-septate, dark brown when mature	ITS, LSU, SSU, *TEF*	*Cotoneaster franchetii*	China	[49], this study
*R. chromolaenae*	500–1000 µm long × 250–500 µm high, superficial, not perpendicular striae, scattered, dark brown to black with dark orange at the center	45–60(–110) µm wide, hyaline or pale brown to brown cells arranged in *textura globulosa* to *textura angularis*	Septate, branched pseudoparaphyses	120–140 µm × 10–15 µm, 8-spored	18–22 µm × 7–9 µm, 3-septate, pale brown to brown when mature	ITS, LSU, SSU, *TEF*	*Chromolaena odorata*	Thailand	[48]
*R. coffeae*	1520 µm long × 1120 µm wide × 450 µm high, rough, solitary to aggregated, mostly solitary, superficial, perpendicular striae, reddish brown at the center	70–160 µm wide, composed of dark brown, thick-walled cells of *textura angularis*, outer layer brown to dark brown, inner layer pale brown to hyaline	Septate, branched, cellular pseudoparaphyses, forming a red to purple epithecium above asci when mounted in water	179.5 µm × 13 µm, 8-spored, J- apical ring	26 µm × 10 µm, 3-septate, reddish brown to brown when mature	ITS, LSU, SSU, *TEF*	*Coffea* sp.	China	This study
*R. columbiense*	1500–3000 µm long × 1200–1800 µm wide × 600–700 µm high, superficial, striated, yellowish green on the margins	60–90 µm wide, dark brown to black, thick-walled cells of *textura angularis*	Septate, branched pseudoparaphyses	175–190 µm × 14–18 µm, 6–8-spored	38–52 µm × 13–18 µm, (1–)3-septate, reddish brown when mature	—	Unidentified woody	Colombia	[27]
*R. cozumelense*	2500–3500 µm long × 1100–1500 µm wide × 800–1900 µm high, erumpent, solitary, smooth to slightly striated, dark at the center	Two layers, the first carbonaceous, 45–100 µm wide thick cells of *textura prismatica*. The second cells hyaline, thin-walled	Septate pseudoparaphyses	182–191 µm × 12–13 µm, 8-spored	26–29 µm × 9–11 µm, 3-septate, dark brown when mature	ITS, LSU, *TEF*	*Tabebuia rosea*	Mexico	[24]
*R. discolor*	1000–2000 µm long, cracking after maturity	Carbonaceous	Paraphyses filiform	180–220 µm × 12–15 µm, 8-spored	28–30 µm × 10–12 µm, 3-septate, elongated ellipse, guttules	—	*Celtis tala*	Argentina	[72]
*R. erioi*	600–1200 µm long × 270–360 µm high, superficial or slightly erumpent, dentate	Ectal excipulum 55–75 µm wide, thin-walled, dark brown cells of *textura angularis* to *textura globulosa*. Medullary excipulum 14–20 µm wide, hyaline cells of *textura porrecta*	Paraphyses septate, slightly branched at the base	140–200 µm × 9–16 µm, 8-spored, J- apical ring	22–28 µm × 9–11 µm, 3-septate, dark brown when mature	ITS, LSU, *TEF*	Unidentified wood	Thailand	[49]
*R. esperanzae*	2000–4500 µm long × 1200–3000 µm wide × 1000–2400 µm high, superficial, solitary, rarely gregarious, margin greyish green, striated, dark green to black at the center	Exciple in two layers, the first carbonaceous, 60–220 µm wide cells of *textura globulosa-angularis*. The second slightly pigmented to hyaline, thin-walled	Septate pseudoparaphyses	265–270 µm × 19–20 µm, 8-spored	45–47 µm × 17–19 µm, 3-septate, reddish brown to brown when mature	ITS, LSU, *TEF*	*Oreomunnea mexicana*	Mexico	[24]
*R. guaraniticum*	1000–4000 µm long × 700–100 µm wide, superficial	—	—	200 µm × 12–14 µm	30–31 µm × 10–12 µm, 3-septate	—	On bark, rotten branches	Jawa, Paraguay	[73]
*R. hongheense*	1200–2000 µm long × 600–1000 µm wide × 350–500 µm high, slightly erumpent, slightly dentate	Ectal excipulum 70–100 µm wide, thick-walled, with black cells of *textura globulosa* to *textura angularis*. Medullary excipulum is composed of narrow, long, thin-walled, hyaline to brown cells of *textura* * angularis*	Septate, branched pseudoparaphyses, forming a red epithecium above asci when mounted in water	140–180 µm × 12–16 µm, 8-spored	20–33 µm × 9–13 µm, 3-septate, dark brown when mature, rarely muriform, with one longitudinal septum	ITS, LSU, SSU, *TEF*, *RPB2*	*Dodonaea* sp., *Phyllanthus emblica*	China	[25], this study
*R. indicum*	1800–3000 µm long, black, carbonaceous, scattered, erumpent, uniloculate, discoid to elongated	—	Paraphyses filiform, septate, clavate expansion	200–220 µm × 18–20 µm, 8-spored	30–32 µm × 12–14 µm, dark brown, 3-septate, end cells slightly tapering, constricted at septa, uniseriate	—	*Scutia indica*	India	[74]
*R. magnoliae*	1200–2300 µm long × 540–600 µm wide × 430–550 µm high µm semi-immersed to superficial, striated, dark brown at the center	80–100 µm wide, two layers. Outer layer black to dark brown, thick-walled cells of *textura angularis*. Inner layer hyaline, thin-walled cells of *textura angularis* to *textura prismatica*	Septate, branched pseudoparaphyses, forming an orange epithecium above asci when mounted in water	160–200 µm × 14 µm, 8-spored	25–32 µm × 8–12 µm, 1–3-septate, pale brown to dark brown when mature	ITS, LSU, SSU, *TEF*	*Hevea brasiliensis,* *Magnolia grandiflora*	China	[6], this study
*R. mangrovei*	930–1980 µm long × 780–910 µm wide × 500–520 µm high, crowded to aggregate, semi-immersed to superficial, rough-striated	65–90 µm wide, dark brown to black, thin-walled cells of *textura angularis*	Septate, unbranched pseudoparaphyses	110–150 µm × 9.4–10 µm, (2–6–) 8-spored	21–28 µm × 7.5–8.5 µm, 1–3-septate, reddish brown when mature	ITS, LSU, *TEF*	*Mangrove* sp.	Thailand	[12]
*R. mengziense*	1400 µm long × 910 µm wide × 640 µm high, smooth, solitary to aggregated, mostly solitary, semi-immersed to superficial, perpendicular striae, reddish brown at the center	60–135 µm wide, composed of outer layer brown to black, thick-walled cells of *textura angularis* and inner layer light brown, thin-walled cells of *textura prismatica*	Septate, branched, cellular pseudoparaphyses, forming a reddish brown to brown epithecium above asci when mounted in water	164.5 µm × 13 µm, 8-spored, J- apical ring	27 µm × 12 µm, 3-septate, reddish brown to brown when mature	ITS, LSU, SSU, *TEF*	*Crataegus scabrifolia*	China	This study
*R. mesophilum*	2500–4000 µm long × 1000–1500 µm wide × 1400–1700 µm high, superficial or erumpent, gregarious, rarely solitary, margin yellowish green, striated, orange at the center	Two layers, the first carbonaceous, 62.5–75 µm wide, green yellowish cells of *textura prismatica*. The second hyaline, thin-walled	Aseptate, branched pseudoparaphyses	267–282 µm × 15.5–16 µm, 8-spored	40–44 µm × 12–14 µm, 3-septate, light brown when mature	ITS, LSU, *TEF*	On decayed wood	Mexico	[24]
*R. mexicanum*	2000–4000 µm long × 1500–2500 µm wide × 1500 µm high, superficial or erumpent, gregarious, rarely solitary, striated	Two layers, the first carbonaceous, 104.5–114 µm wide in the medium cells of *textura globulosa* to *textura angularis*, thick-walled. The second composed of cells of *textura* * prismatica*, hyaline, thin-walled	Aseptate, bifurcated to branched pseudoparaphyses	285–297 µm × 16–17 µm, 8-spored	34–40 µm × 10–12 µm, 3-septate, reddish brown when mature	ITS, LSU	—	Mexico	[51]
*R. neohysterinum*	1500–2500 µm long × 700–2200 µm wide × 700–1100 µm high, superficial, solitary, rarely gregarious, striated, orange at the center	52–68 µm wide, dark brown to black, thick-walled cells of *textura prismatica*	Septate pseudoparaphyses	160–185 µm × 12–13 µm, 8-spored	24.8–29 µm × 8.8–10 µm, 1-septate, brown when mature	—	*Acacia* sp.	Mexico	[55]
*R. neorufulum*	835–2100 µm long × 350–1320 µm wide × 430–1000 µm high, superficial, elliptic or irregular, without striations, black or yellow at the center	64–160 µm wide, dark brown to black, thick-walled cells of *textura angularis*	Septate, branched pseudoparaphyses, forming a yellow epithecium above asci when mounted in water	185–260 µm × 9.5–18 µm, 8-spored	27–44 µm × 6.5–17 µm, 1–3-septate, reddish brown to brown when mature	ITS, LSU, SSU, *TEF*	*Bursera* sp., *Elaeagnus sarmentosa,* Hevea brasiliensis, Tectona grandis	China, Mexico, Thailand	[16,18,55], this study
*R. opuntiae*	640–1700 µm long	—	—	85–160 µm × 12.5–16 µm, 3–8-spored	17–33 µm × 13 µm, 3–5-septate	LSU, *TEF*	*Opuntia fulgida*	USA	[52]
*R. prosopidis*	Superficial, very hard when dry, elliptical or triangular, black, with very obtuse, thick, yellowish green disc	—	—	—	3-septate, unineriate, oblong, sometimes slightly curved	—	*Prosopis juliflora*	USA	[75]
*R. quercinum*	1000–3000 µm in diam., leathery apothecia, scattered, superficial, erumpent, pedicellate (short pedicel)	Excipulum black with low seated, reddish	—	Asci cylindrical, slender, stalked hyaline with inconspicuous wall	19.0–24.7 µm × 7.6–11.4 µm, 1–3-septate more commonly 3	—	*Quercus* sp.	India	[76]
*R. tectonae*	550–3365 µm long × 325–728 µm wide × 370−835 µm high, semi-immersed to superficial, smooth without striation, yellow at the center	80–135 µm wide, two layers. Outer layer black to dark reddish, thick-walled cells of *textura angularis*. Inner layer hyaline, thin-walled cells of *textura angularis*	Septate, branched pseudoparaphyses, forming an orange epithecium above asci when mounted in water	150–200 µm × 10–15 µm, 8-spored	19–31 µm × 8–13 µm, 1–3-septate, pale brown to dark brown when mature	ITS, LSU, SSU, *TEF*	*Betula* sp., Fabaceae sp., *Magnolia delavayi,* Tectona grandis	China, Thailand	[18,53], this study
*R. viride*	1000–1500 µm long × 500–600 µm wide, erumpent	—	Filiform, hyaline	200–250 µm × 10–12 µm	20–30 µm × 7–9 µm, 3-septate	—	On bark, associated with lichens	Brazil	[77]
*R. yunnanense*	2510 µm × 625 µm × 455 µm, solitary to aggregated, mostly aggregated, semi-immersed, each hysterothecia has two parallel striae parallel to the longitudinal slit and slight perpendicular striae, longitudinal slit, no opening	60–180 µm wide, composed of dark brown, thick-walled cells of *textura globulosa*, outer layer brown to dark brown, inner layer pale brown to hyaline	Septate, branched, cellular pseudoparaphyses, forming a yellow epithecium above asci when mounted in water	230 µm × 14 µm, 8-spored, J- apical ring	32.5 µm × 13 µm, 3-septate, reddish brown to brown when mature	ITS, LSU, SSU, *TEF*	*Rhus chinensis*	China	This study

**Remarks:** the symbol “—” denotes no information available.

## Data Availability

Not applicable.

## Supplementary Materials

The following supporting information can be downloaded at: https://www.mdpi.com/article/10.3390/jof9020148/s1, Supplementary Notes S1–S7: Description of seven new records; Supplementary Figures S1–S7: Photo plates of seven new records.
