# Supplementary material for: Additions to Rhytidhysteron (Hysteriales, Dothideomycetes) in China
Source: J Fungi (Basel). 2023 Jan 22;9(2):148. doi: 10.3390/jof9020148 (PMC9958654; doi:10.3390/jof9020148)
Supplement: Supplementary file 1 [file jof-09-00148-s001.zip › jof-2143901-supplementary.pdf]

Journal of Fungi

MDPI

Supplementary

Supplementary notes S1

Rhytidhysteron bruguierae Dayarathne, Mycosphere 11(1): 20 (2020) (Figure S1)

Index Fungorum number: IF 556574. Facesoffungi number: FoF 06154

Saprobic on decaying wood of *Alnus nepalensis* (Betulaceae). Sexual morph: *Ascomata* 1000–1600 µm long × 600–1110 µm wide × 400–600 µm high ($\bar{x}$ = 1450 × 850 × 520 µm, n = 5), hysterothecial, solitary, superficial, base is embedded in the plant tissue, navicular, black, apothecioid, rough, perpendicularly striate, compressed at apex, and hysterothecia open a nearly circular opening through a longitudinal slit, folded along the margins when dry, orange at the center. *Exciple* 90–120 µm wide, composed of outer layer dark brown to black, thick-walled cells of *textura angularis*, and inner layer hyaline, thick-walled cells of *textura globulosa*. *Hamathecium* comprising 1–2 µm wide, dense, hyaline, septate, branched, cellular pseudoparaphyses, forming a red epithecium above asci when mounted in water, becoming purple epithecium above the asci when mounted in 10% KOH and turns hyaline after 30 seconds. *Asci* (127–)130–165(–173) µm × (11.5–)13–15 µm ($\bar{x}$= 155 × 13.8 µm, n = 20), 8-spored, bitunicate, cylindrical, with short pedicel, rounded at the apex, with an ocular chamber, J- apical ring. *Ascospores* 20–24 µm × (7.8–)9–11 µm ($\bar{x}$= 22 × 10 µm, n = 30), uniseriate, hyaline, 1-septate when immature, becoming brown, 3-septate when mature, ellipsoidal to fusiform, rounded to slightly pointed at both ends, guttulate, smooth-walled, without the mucilaginous sheath. Asexual morph: Undetermined.

Culture characteristics: Ascospores germinating on PDA within 24 h and germ tubes produced from one or both ends. Colonies on PDA reached a 6 cm diameter after two weeks at 28°C. The colony was soft, circular, slightly raised, with a filiform edge, white on the forward and reverse.

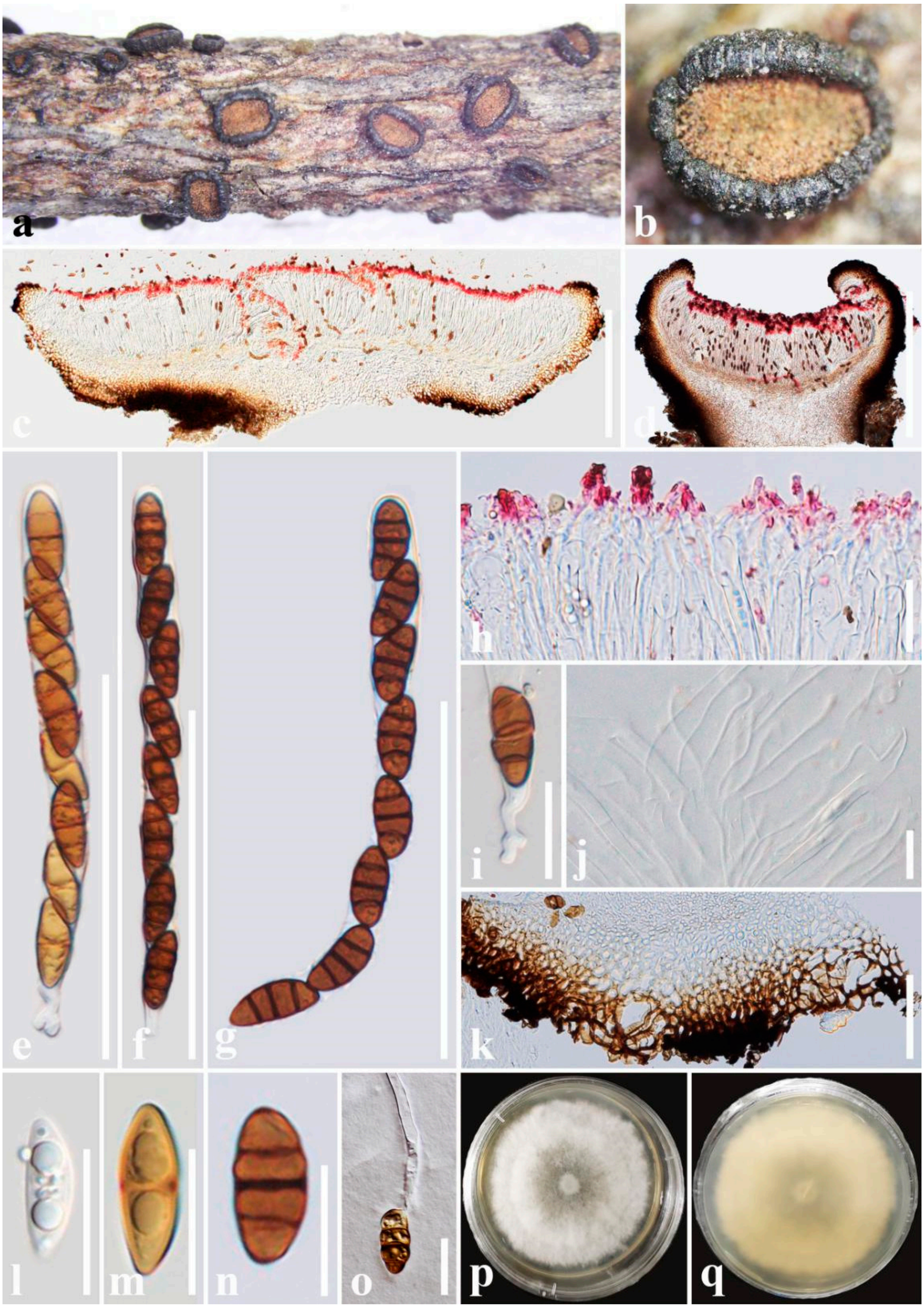

Figure S1. ***Rhytidhysteron bruguierae*** (HKAS 122690). (**a, b**) Appearance of hysterothecia on the host; (**c, d**) Vertical section through hysterothecium; (**e-g**) Asci; (**h**) Epithecium mounted in water; (**i**) Pedicel of asci; (**j**) Pseudoparaphyses; (**k**) Exciple; (**l-n**) Ascospores; (**o**) A germinating ascospore; (**p, q**) Colony on PDA medium (after one week). Scale bars: (**c, d**) = 500 μm; (**e-g**) = 100 μm; (**h-j, l-o**) = 20 μm; (**k**) = 50 μm.

Supplementary notes S2

Rhytidhysteron camporesii Ekanayaka & K.D. Hyde, Fungal Diversity 100: 5–277 (2020) (Figure S2)

Index Fungorum number: IF 556783. Facesoffungi number: FoF 06459

Saprobic on decaying wood of *Cotoneaster franchetii* (Rosaceae). Sexual morph: *Ascomata* 1200–1600 µm long × 900–1200 µm wide × 450–650 µm high ($\bar{x}$ = 1430 × 1050 × 530 µm, n = 5), hysterothecial, solitary, superficial, base is embedded in the plant tissue, navicular, black, apothecioid, smooth, perpendicularly striate, elongate and depressed, compressed at apex, and open a nearly circular opening through a longitudinal slit, dark reddish-brown at the center. *Exciple* 50–135 µm wide, composed of dark brown, thick-walled cells of *textura angularis*, outer layer brown to dark brown, inner layer pale brown to reddish-brown. *Hamathecium* comprising 1–2 µm wide, dense, hyaline, septate, branched, cellular pseudoparaphyses, forming an orange-red epithecium above asci when mounted in water, becoming purple epithecium above the asci when mounted in 10% KOH and turns hyaline after 30 seconds. *Asci* (147–)155–225 µm × 13.7–17.7(–18.5) µm ($\bar{x}$= 177 × 15 µm, n = 20), 8-spored, bitunicate, cylindrical, with short pedicel, rounded at the apex, with an ocular chamber, J- apical ring. *Ascospores* (24–)27–33 µm × 10–12.5 µm ($\bar{x}$= 28 × 11 µm, n = 30), uni-seriate, hyaline, 1-septate when immature, becoming brown, 3-septate when mature, ellipsoidal to fusiform, straight or curved, rounded to slightly pointed at both ends, guttulate, smooth-walled, without a mucilaginous sheath. Asexual morph: Undetermined.

Culture characteristics: Ascospores germinating on PDA within 24 h and germ tubes produced from one or both ends. Colonies on PDA reached a 6 cm diameter after two weeks at 28°C. The colony was soft, circular, slightly raised, with a filiform edge, reddish-brown to dark grey on the forward and yellowish-brown in reverse.

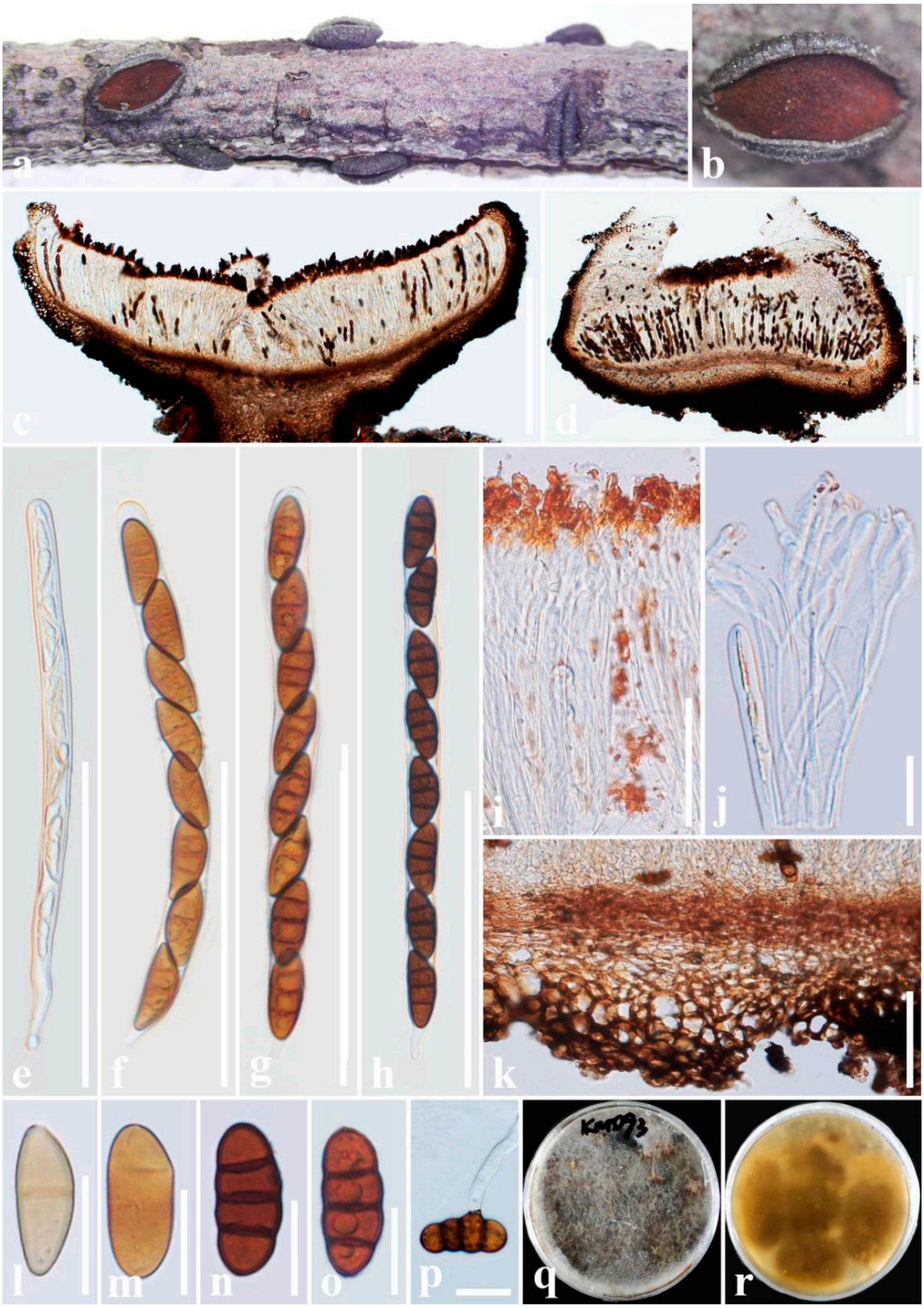

Figure S2. ***Rhytidhysteron camporesii*** (HKAS 122698). (**a, b**) Appearance of hysterothecia on the host; (**c, d**) Vertical section through hysterothecium; (**e-h**) Asci; (**i**) Epithecium mounted in water; (**j**) Pseudoparaphyses; (**k**) Exciple; (**l-o**) Ascospores; (**p**) A germinating ascospore; (**q, r**) Colony on PDA medium (after four weeks). Scale bars: (**c, d**) = 500 µm; (**e-h**) = 100 µm; (**i, k**) = 50 µm; (**j, l-p**) = 20 µm.

Supplementary notes S3

Rhytidhysteron hongheense Wanas. J. Fungi 7, 180 (2021) (Figure S3)

Index Fungorum number: IF 837992

Saprobic on decaying wood of *Phyllanthus emblica* (Euphorbiaceae). Sexual morph: *Ascomata* 900–1100 µm long × 750–900 µm wide × 350–550 µm high ($\bar{x}$ = 955 × 866 × 440 µm, n = 5), hysterothecial, solitary to aggregated, mostly solitary, semi-immersed to superficial, navicular, black, apothecioid, rough, slightly perpendicularly striate, elongate and depressed, compressed at apex, longitudinal slit no opening. *Exciple* 50–130 µm wide, composed of dark brown, thick-walled cells of *textura angularis*, outer layer brown to dark brown, inner layer pale brown. *Hamathecium* comprising 1–2 µm wide, dense, hyaline, septate, branched, cellular pseudoparaphyses, forming a red epithecium above asci when mounted in water, becoming purple epithecium above the asci when mounted in 10% KOH and turns hyaline after 30 seconds. *Asci* 163–200 µm × 12.5–16 µm ($\bar{x}$= 183.5 × 14 µm, n = 20), 8-spored, bitunicate, cylindrical, with short pedicel, rounded at the apex, with an ocular chamber, J- apical ring. *Ascospores* 25–27 µm × 9.5–10.5 µm ($\bar{x}$= 26 × 10 µm, n = 30), uni-seriate, slightly overlapping, hyaline, 1-septate when immature, becoming brown, 1–3-septate when mature, ellipsoidal to fusoid, straight or curved, rounded to slightly pointed at both ends, guttulate, smooth-walled, without a mucilaginous sheath. Asexual morph: Undetermined.

Culture characteristics: Ascospores germinating on PDA within 24 h and germ tubes produced from one or both ends. Colonies on PDA reached a 6 cm diameter after two weeks at 28°C. The colony was soft, circular, slightly raised, with an entire edge, white to grey on the forward and dark grey in reverse.

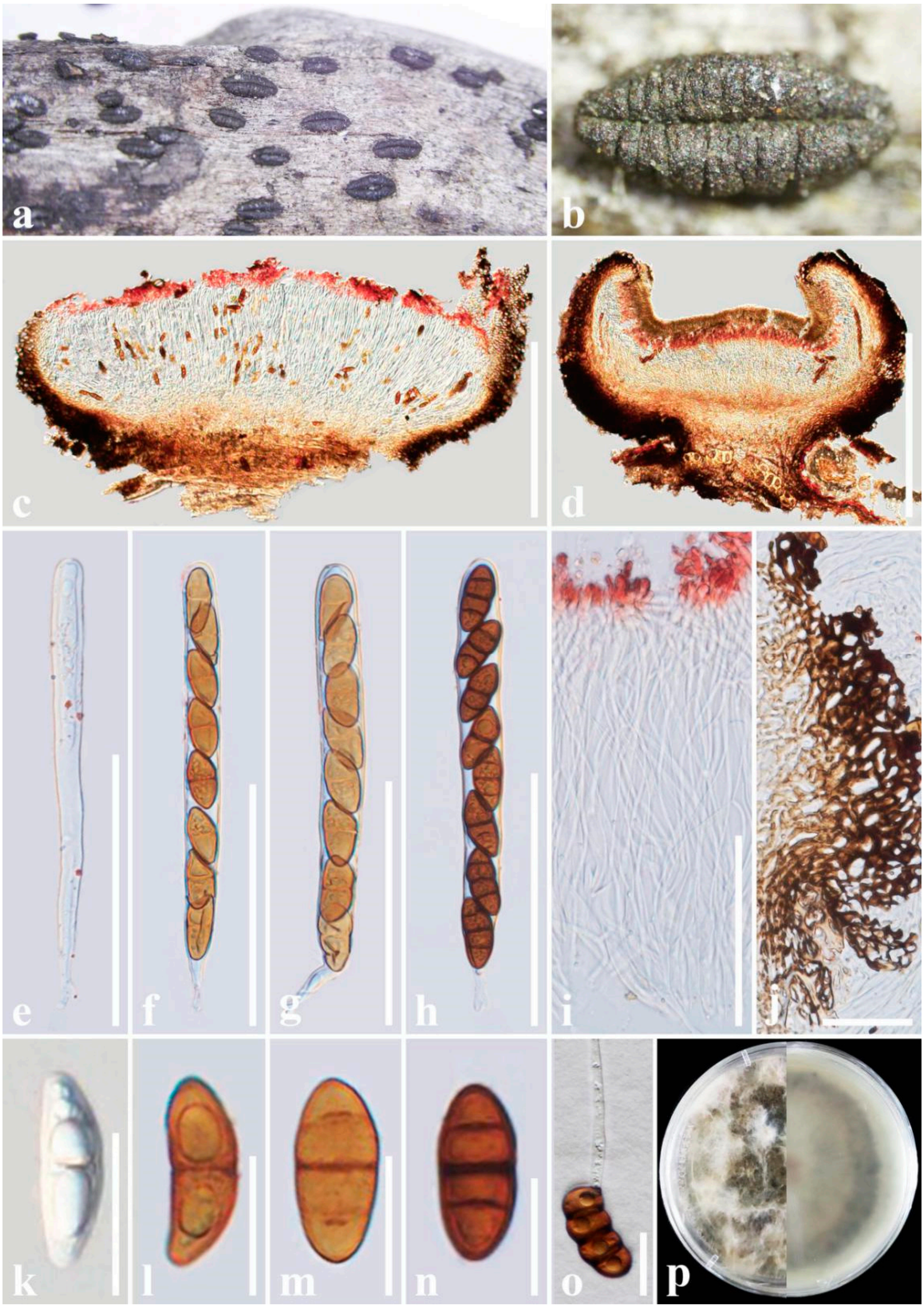

Figure S3. ***Rhytidhysteron hongheense*** (HKAS 122698, paratype). (**a, b**) Appearance of hysterothecia on the host; (**c, d**) Vertical section through hysterothecium; (**e-h**) Asci; (**i**) Epithecium and pseudoparaphyses mounted in water; (**j**) Exciple; (**k-n**) Ascospores; (**o**) A germinating ascospore; (**p**) Colony on PDA medium (after four weeks). Scale bars: (**c, d**) = 500 μm; (**e-i**) = 100 μm; (**j**) = 50 μm; (**k-o**) = 20 μm.

Supplementary notes S4

Rhytidhysteron magnoliae N.I. de Silva, Lumyong S & K.D. Hyde, Asian Journal of Mycology 3(1): 295–306 (2019) (Figure S4)

Index Fungorum number: IF 557220. Facesoffungi number: FoF 07369

Saprobic on decaying wood of *Hevea brasiliensis* (Euphorbiaceae). Sexual morph: *Ascomata* 1800–2200 μm long × 450–1000 μm wide × 450–900 μm high ($\bar{x}$ = 2015 × 750 × 660 μm, n = 5), hysterothecial, solitary to aggregated, mostly aggregated, semi-immersed to superficial, fusiform or irregular in shape, coriaceous, navicular to irregular, black, apothecioid, slightly perpendicularly striate, elongate and depressed, compressed at apex, seldom opening by a longitudinal slit, orange at the center. *Exciple* 40–130 μm wide, composed of outer layer dark brown to black, dense, thick-walled cells of *textura angularis*, and inner layer light brown, thin-walled cells of *textura globulosa*. *Hamathecium* comprising 1–2 μm wide, dense, hyaline, septate, branched, cellular pseudoparaphyses, slightly swollen at the apex and enclosed in a gelatinous matrix, forming an orange epithecium above asci when mounted in water, becoming purple epithecium above the asci when mounted in 10% KOH and turns hyaline after 5 seconds. *Asci* 170–240 μm × (11–)13–17 μm ($\bar{x}$ = 202.5 × 15 μm, n = 20), 8-spored, bitunicate, cylindrical, with short pedicel, rounded at the apex, with an ocular chamber, J- apical ring. *Ascospores* 26–32(–25) μm × 11–14 μm ($\bar{x}$ = 30 × 13 μm, n = 30), uni-seriate, slightly overlapping, hyaline, 1-septate when immature, becoming brown to dark brown, 1–3-septate when mature, ellipsoidal to fusiform, straight or curved, rounded to slightly pointed at both ends, guttulate, smooth-walled, without a mucilaginous sheath. Asexual morph: Undetermined.

Culture characteristics: Ascospores germinating on PDA within 24 h and germ tubes produced from one or both ends. Colonies on PDA reached a 6 cm diameter after two weeks at 28°C. The colony was soft, irregular, slightly raised, with an undulated edge, grey on the forward and yellowish-brown in reverse.

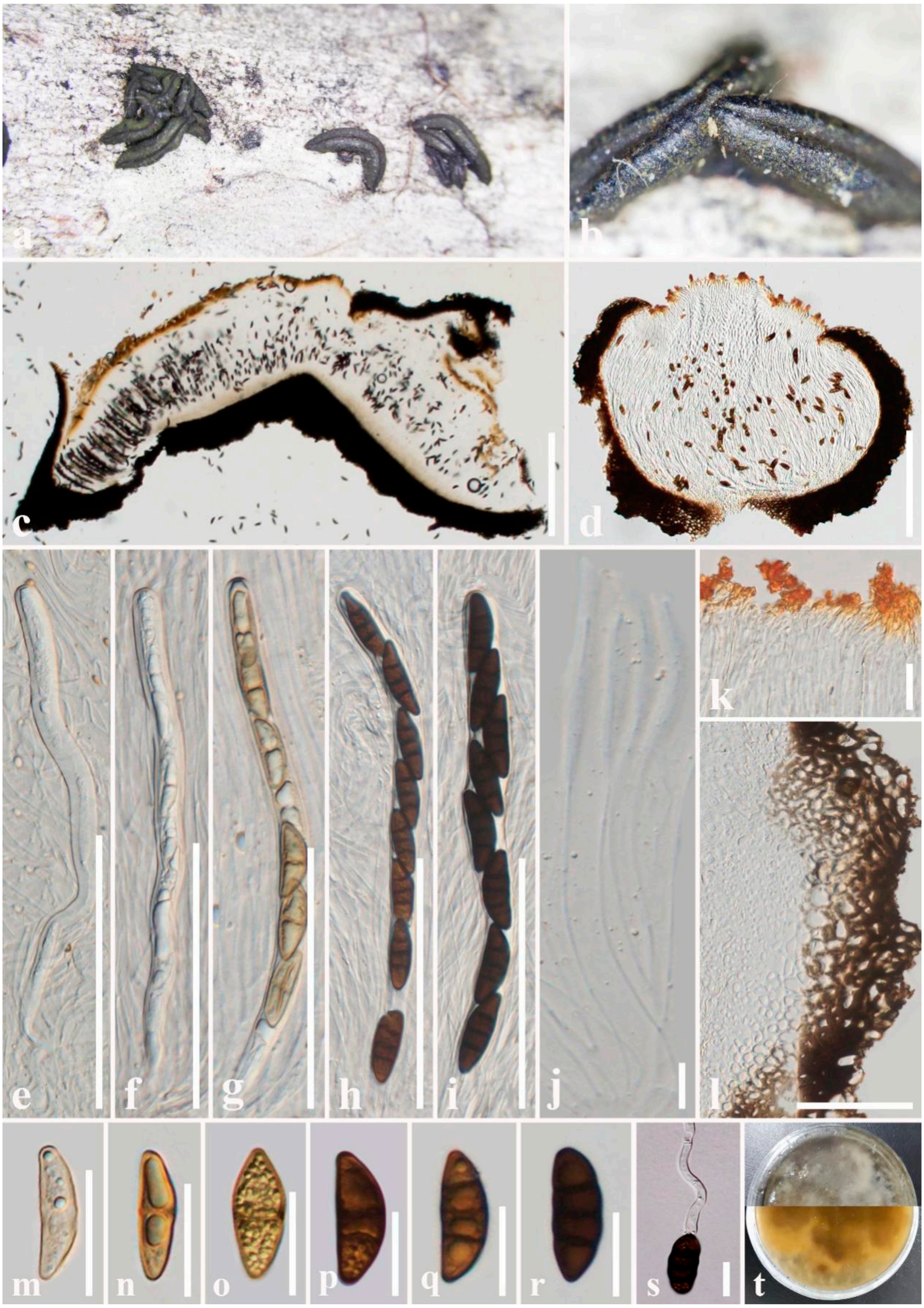

Figure S4. ***Rhytidhysteron magnoliae*** (HKAS 122693). (**a, b**) Appearance of hysterothecia on the host; (**c, d**) Vertical section through hysterothecium; (**e-i**) Asci; (**j**) Pseudoparaphyses; (**k**) Epithecium mounted in water; (**l**) Exciple; (**m-r**) Ascospores; (**s**) A germinating ascospore; (**t**) Colony on PDA medium (after four weeks). Scale bars: (**c**) = 500 μm; (**d**) = 200 μm; (**e-i**) = 100 μm; (**j, k, m-s**) = 20 μm; (**l**) = 50 μm.

Supplementary notes S5

Rhytidhysteron neorufulum Thambug. & K.D. Hyde, Cryptog. Mycol. 37(1): 110

(2016) (Figure S5)

Index Fungorum number: IF 551865. Facesoffungi number: FoF 01840

Saprobic on decaying wood of *Elaeagnus sarmentosa* (Elaeagnaceae). Sexual morph: *Ascomata* 1000–1200 μm long × 750–1000 μm wide × 350–650 μm high ($\bar{x}$ = 1115 × 850 × 510 μm, n = 5), hysterothecial, solitary to aggregated, superficial, base is embedded in the plant tissue, navicular, black, apothecioid, rough, without striate, elongate and depressed, compressed at apex, longitudinal slit no opening. *Exciple* 35–80 μm wide, composed of dark brown, thick-walled cells of *textura angularis* to *textura globulosa*, outer layer brown to dark brown, inner layer pale brown to hyaline. *Hamathecium* comprising 1–2.5 μm wide, dense, hyaline, septate, branched, cellular pseudoparaphyses, forming a yellow epithecium above asci when mounted in water, becoming purple epithecium above the asci when mounted in 10% KOH and turns hyaline after 5 seconds. *Asci* 185–257 μm × (13–)14–18 μm ($\bar{x}$= 218 × 15.5 μm, n = 20), 8-spored, bitunicate, cylindrical, with short pedicel, rounded at the apex, with an ocular chamber, J- apical ring. *Ascospores* (28–)30–34 μm × 13–15.5 μm ($\bar{x}$= 32.5 × 14 μm, n = 30), uni-seriate, slightly overlapping, hyaline, 1-septate when immature, becoming brown to dark brown, 3-septate when mature, ellipsoidal to fusiform, straight or curved, rounded to slightly pointed at both ends, guttulate, smooth-walled, without a mucilaginous sheath. Asexual morph: Undetermined.

Culture characteristics: Ascospores germinating on PDA within 24 h and germ tubes produced from one or both ends. Colonies on PDA reached a 6 cm diameter after two weeks at 28°C. The colony was soft, irregular, slightly raised, with an undulated edge, white on the forward and reverse.

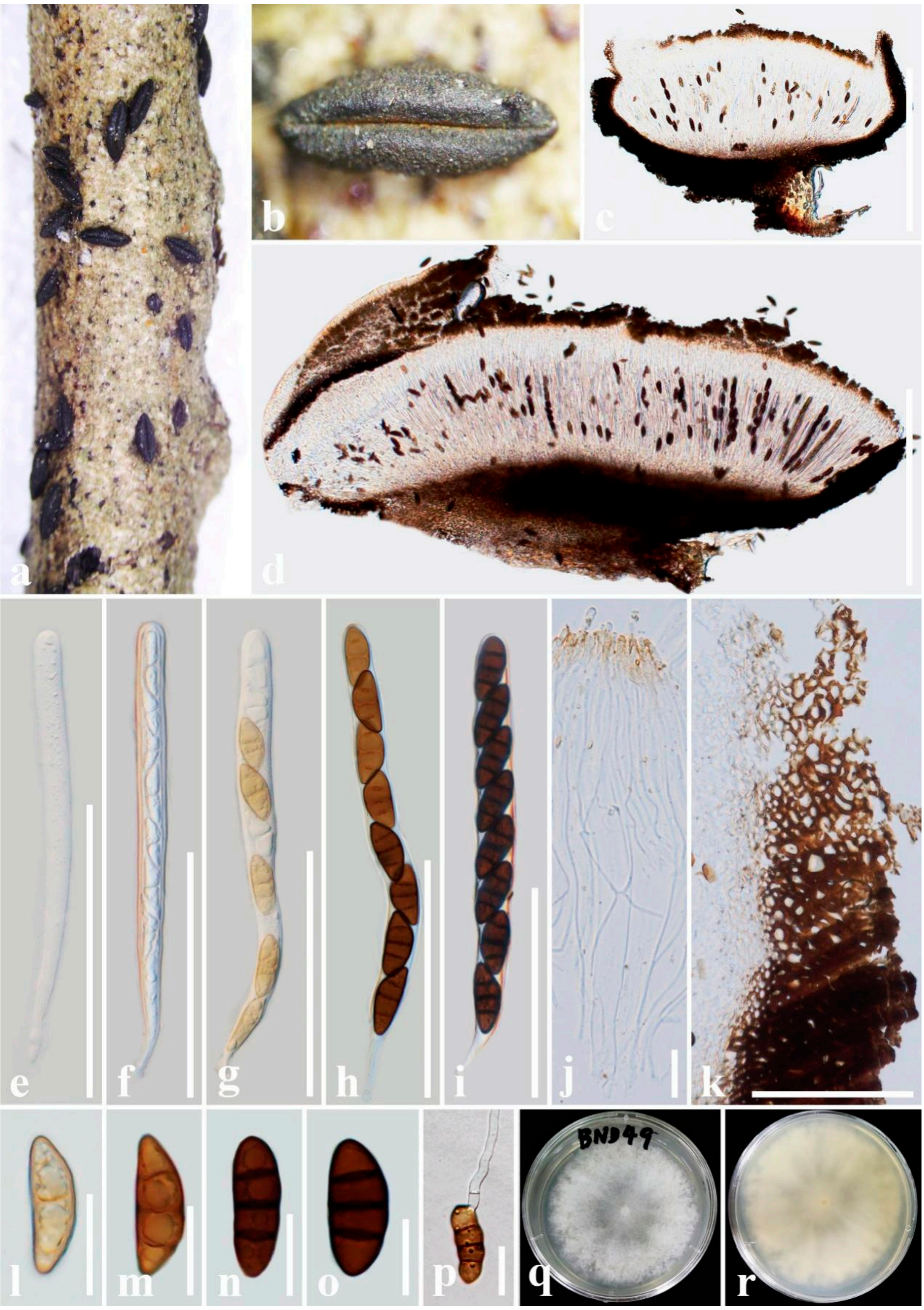

Figure S5. ***Rhytidhysteron neorufulum*** (HKAS 122691). (**a, b**) Appearance of hysterothecia on the host; (**c, d**) Vertical section through hysterothecium; (**e-i**) Asci; (**j**) Epithecium and pseudoparaphyses mounted in water; (**k**) Exciple; (**l-o**) Ascospores; (**p**) A germinating ascospore; (**q, r**) Colony on PDA medium (after one week). Scale bars: (**c, d**) = 500 µm; (**e-i**) = 100 µm; (**j, l-p**) = 20 µm; (**k**) = 50 µm.

Supplementary notes S6

Rhytidhysteron tectonae Doilom & K.D. Hyde, Fungal Diversity. 82: 107–182 (2017) (Figure S6)

Index Fungorum number: IF 551964. Facesoffungi number: FoF 01849

Saprobic on decaying wood of *Magnolia delavayi* (Magnoliaceae). Sexual morph: *Ascomata* 1800–2300 μm long × 450–800 μm wide × 450–1000 μm high ($\bar{x}$ = 1950 × 605 × 650 μm, n = 5), hysterothecial, solitary to aggregated, mostly solitary, semi-immersed to superficial, navicular, black, apothecioid, rough, without striate, elongate and depressed, compressed at apex, opening by a small longitudinal slit, yellow at the center. *Exciple* 60–150 μm wide, composed of outer layer yellow to brown, thick-walled cells of *textura angularis*, and inner layer hyaline, thin-walled cells of *textura globulosa*. *Hamathecium* comprising 1–2 μm wide, dense, hyaline, septate, branched, cellular pseudoparaphyses, forming an orange epithecium above asci when mounted in water, becoming purple epithecium above the asci when mounted in 10% KOH and turns hyaline after 5 seconds. *Asci* (176–)184–233 μm × 12–17 μm ($\bar{x}$= 202 × 14 μm, n = 20), 8-spored, bitunicate, cylindrical, with short pedicel, rounded at the apex, with an ocular chamber, J- apical ring. *Ascospores* (25–)27–34 μm × (7–)8–10 μm ($\bar{x}$= 30 × 9 μm, n = 30), uni-seriate, slightly overlapping, hyaline, 1-septate when immature, becoming brown to dark brown, 1–3-septate when mature, ellipsoidal to fusoid, straight or curved, rounded to slightly pointed at both ends, guttulate, smooth-walled, without a mucilaginous sheath. Asexual morph: Undetermined.

Culture characteristics: Ascospores germinating on PDA within 24 h and germ tubes produced from one or both ends. Colonies on PDA reached a 4 cm diameter after two weeks at 28°C. The colony was soft, circular, slightly raised, with a filiform edge, light yellow on the forward and brow in reverse.

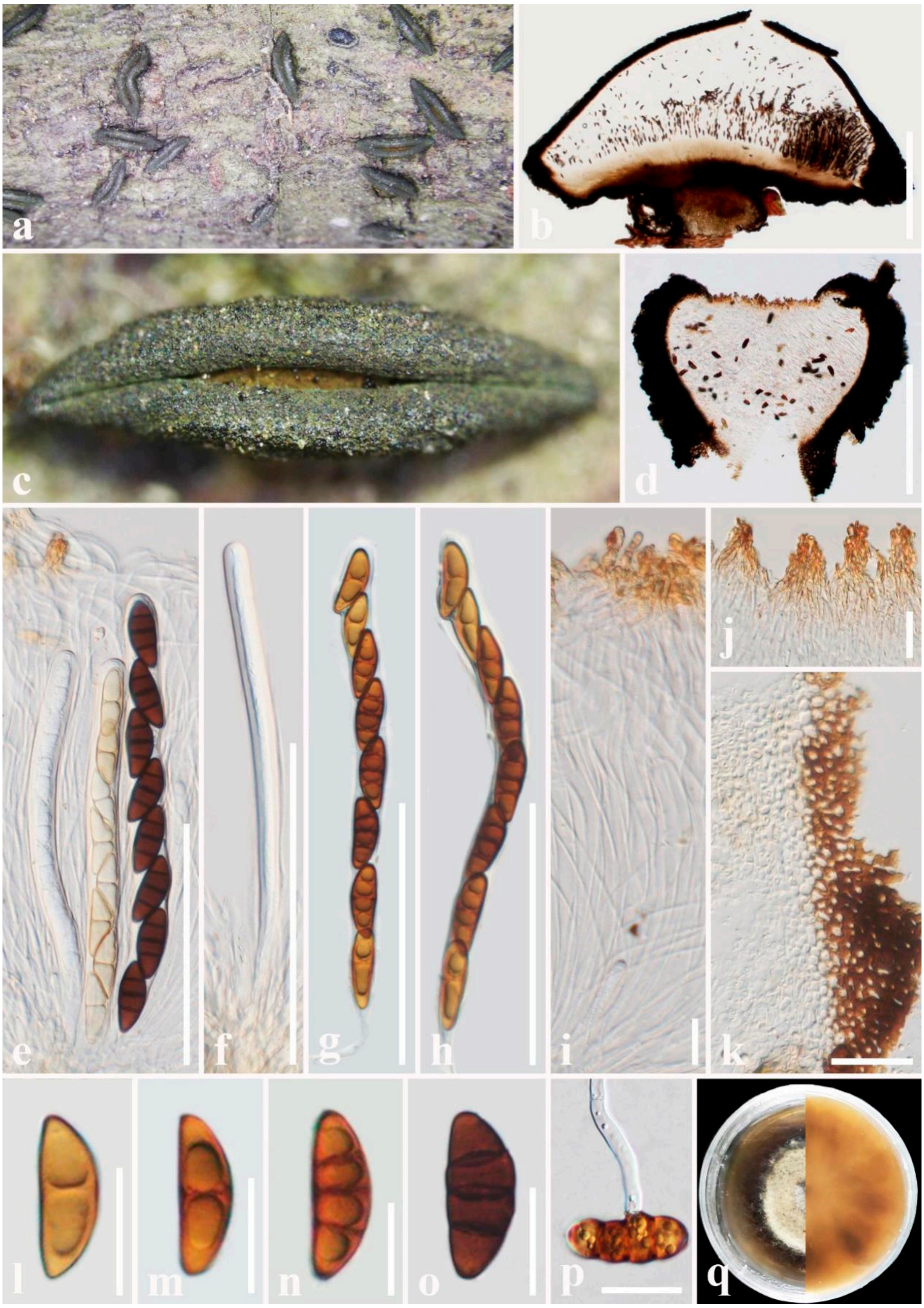

Figure S6. ***Rhytidhysteron tectonae*** (HKAS 122692). (**a, c**) Appearance of hysterothecia on the host; (**b, d**) Vertical section through hysterothecium; (**e-h**) Asci; (**i**) Pseudoparaphyses; (**j**) Epithecium mounted in water; (**k**) Exciple; (**l-o**) Ascospores; (**p**) A germinating ascospore; (**q**) Colony on PDA medium (after four weeks). Scale bars: (**b, d**) = 500 µm; (**e-h**) = 100 µm; (**i, l-p**) = 100 µm; (**j**) = 10 µm; (**k**) = 50 µm.

Supplementary notes S7

Rhytidhysteron thailandicum Thambug. & K.D. Hyde, Cryptog. Mycol. 37(1): 110 (2016) (Figure S7)

Index Fungorum number: IF 551866. Facesoffungi number: FoF 01841

Saprobic on decaying wood of *Aquilaria sinensis* (Thymelaeaceae). Sexual morph: *Ascomata* 800–1300 µm long × 550–700 µm wide × 400–650 µm high ($\bar{x}$ = 1210 × 660 × 580 µm, n = 5), hysterothecial, solitary to aggregated, mostly aggregated, superficial, base is embedded in the plant tissue, navicular, black, apothecioid, slightly perpendicularly striate, surface light green, elongate and depressed, compressed at apex, longitudinal slit no opening. *Exciple* 35–95 µm wide, composed of dark brown, thick-walled cells of *textura angularis*, outer layer, dense, brown to dark brown, inner layer pale brown to hyaline. *Hamathecium* comprising 1–2 µm wide, dense, hyaline, septate, branched, cellular pseudoparaphyses, forming a yellow epithecium above asci when mounted in water, becoming purple epithecium above the asci when mounted in 10% KOH and turns hyaline after 5 seconds. *Asci* 225–246(–250) µm × (15–)16.5–21 µm ($\bar{x}$= 236.5 × 18 µm, n = 20), 8-spored, bitunicate, cylindrical, with short pedicel, rounded at the apex, with an ocular chamber, J- apical ring. *Ascospores* 30–34.5 µm × 11–13.5 µm ($\bar{x}$= 32.5 × 12 µm, n = 30), uniseriate, slightly overlapping, hyaline, 1-septate when immature, becoming dark brown, 3-septate when mature, ellipsoidal to fusoid, straight or curved, rounded to slightly pointed at both ends, guttulate, smooth-walled, without a mucilaginous sheath. Asexual morph: Undetermined.

Culture characteristics: Ascospores germinating on PDA within 24 h and germ tubes produced from one or both ends. Colonies on PDA reached a 6 cm diameter after two weeks at 28°C. The colony was soft, circular, slightly raised, with a filiform edge, white to light brown on the forward and dark grey in reverse.

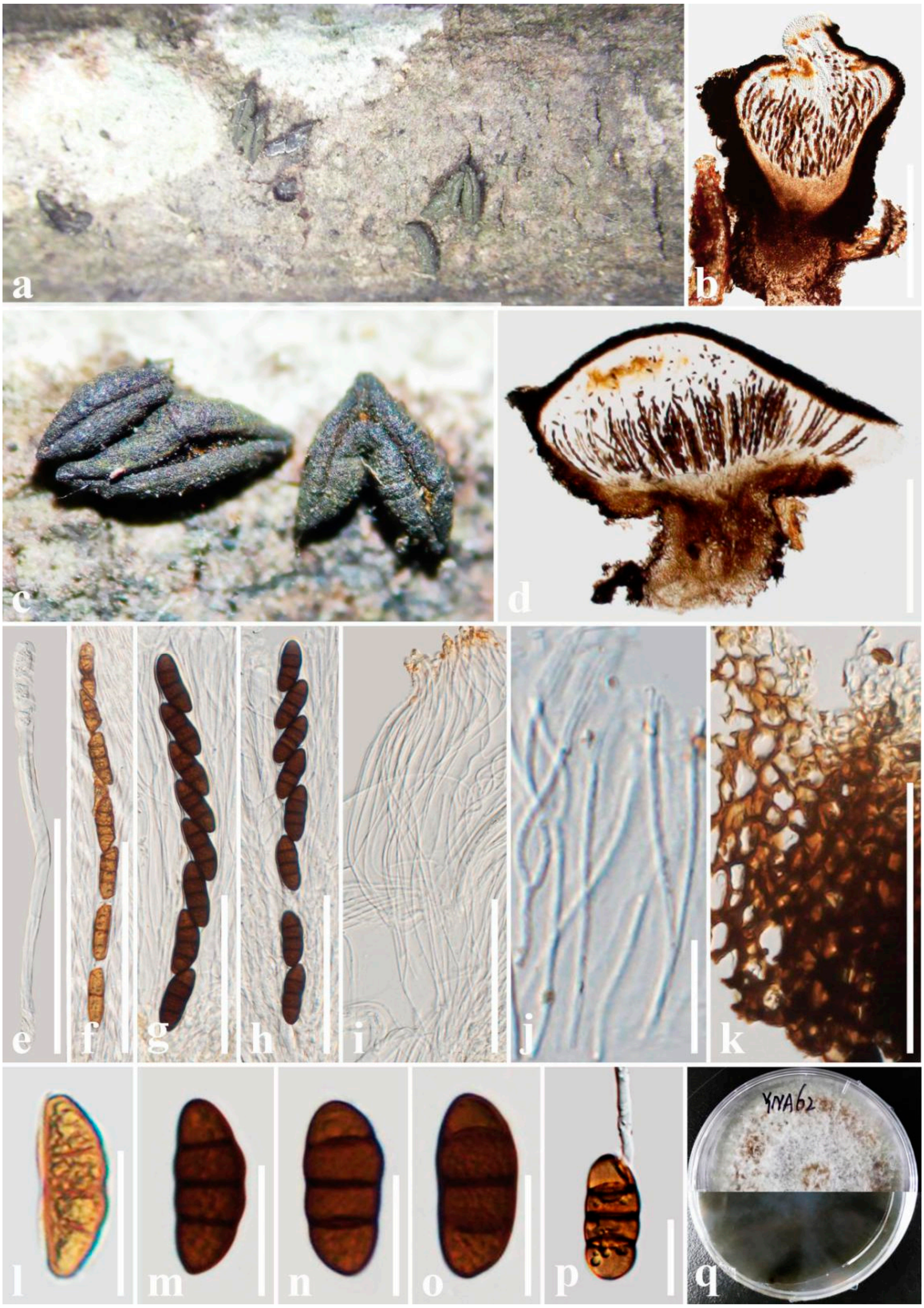

Figure S7. ***Rhytidhysteron thailandicum*** (HKAS 122698). (**a, c**) Appearance of hysterothecia on the host; (**b, d**) Vertical section through hysterothecium; (**e-h**) Asci; (**i, j**) Pseudoparaphyses; (**k**) Exciple; (**l-o**) Ascospores; (**p**) A germinating ascospore; (**q**) Colony on PDA medium (after four weeks). Scale bars: (**b, d**) = 500 μm; (**e-i**) = 100 μm; (**j, l-p**) = 20 μm; (**k**) = 50 μm.
